# Neighbour Effects and Dynamics of Plant‐Associated Microbiomes in a Strip‐Crop System

**DOI:** 10.1111/1758-2229.70423

**Published:** 2026-10-01

**Authors:** Enoch Narh Kudjordjie, Mette Vestergård, Mesfin Tsegaye Gebremikael, Otto Nielsen, Mogens Nicolaisen

**Affiliations:** ^1^ Department of Agroecology, Faculty of Technical Sciences Aarhus University Slagelse Denmark; ^2^ Department of Food Science—Plant, Food & Sustainability, Faculty of Technical Sciences Aarhus University Aarhus Denmark; ^3^ Nordic Beet Research (NBR) Holeby Denmark

**Keywords:** barley microbiome, diverse cropping system, faba bean microbiome, fungal pathogen dynamics, phyllosphere microbiome, plant neighbour effect, soil microbiome

## Abstract

While multi‐cropping systems, such as strip‐cropping, assemble complex microbial communities that contribute to ecosystem resilience, we have limited understanding of their assembly and functions at field scale. Here, we examined the dynamics and site‐specific assembly of soil and phyllosphere microbiomes in a rotational faba bean ( *Vicia faba* ) and spring barley ( *Hordeum vulgare* ) strip‐system during two seasons. Using bacterial 16S and fungal ITS amplicon sequencing, we analysed microbial communities across gradients from the centre towards strip edges. We found that phyllosphere microbiomes are more affected by strip‐cropping than soil microbiomes. Neighbouring crops harbour different microbial communities, but with increasing proximity, their phyllosphere microbiomes share more specific microbial taxa. Likewise, we observed gradient‐specific microbial distribution, including fungal pathogen taxa. For instance, *Puccinia* was more abundant in the centre of the spring barley strips and gradually declined towards the edge. Correlation and co‐occurrence network analyses revealed distinct microbial interaction patterns across soil and phyllosphere gradients. These networks were more resilient at the edge of the strips compared to central networks of spring barley. Altogether, our findings revealed neighbour‐effects modulating host‐associated microbiomes across strips that could be harnessed for designing optimal cropping systems, for example, to reduce leaf pathogens.

## Graphical abstract

Distinct plant species differentially affect the assembly of host‐associated microbial communities in a strip‐crop system. Crop neighbouring effects on the microbiomes were highest at the edge of the strips, which further reveals reduced leaf pathogens.

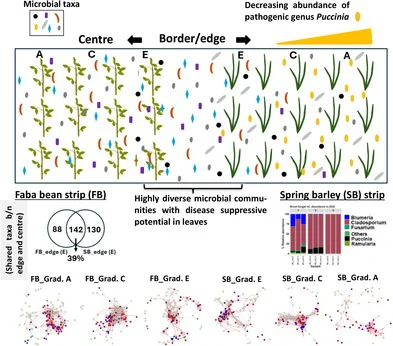

## 1 Introduction

Diversified cropping systems including inter‐cropping and strip‐cropping, where two or more crops are co‐cultivated, have generally been shown to be highly productive and resilient to biotic and abiotic stresses compared to monocropped systems (Alarcón‐Segura et al. 2022; Li et al. 2014; Liu and Yang 2024). Environmental benefits such as improved soil fertility and biodiversity characterizing diversified cropping systems minimizes agrochemical use (Zhang et al. 2025) while maintaining high crop yields and production (Beillouin et al. 2021; Renard and Tilman 2019). Accumulating evidence suggests that these benefits of diversified cropping are driven, at least in part, by the microbial communities, or microbiomes, associated with plants (Jiang et al. 2024; Trinchera et al. 2022; Wang et al. 2021). Plant‐associated microbiomes play a vital role in promoting plant growth by enhancing nutrient acquisition (Custódio et al. 2022; Trivedi et al. 2020), suppressing diseases (Kudjordjie et al. 2024, 2022) and improving overall soil health (Saleem et al. 2019). An increasing number of studies have found that diversified cropping systems assemble more diverse microbiomes than monoculture systems (Jiang et al. 2024), and these diverse host‐associated microbiomes have higher resilience to both biotic and abiotic stresses (Jiang et al. 2024; Zong et al. 2024). While technological advancements and high‐throughput omics techniques are enhancing our understanding of plant‐associated microbiota, the detailed mechanisms underlying plant‐microbiota interactions for sustainable crop production remain to be fully uncovered.

Agricultural fields are highly heterogenous with uneven distribution of microorganisms that are continuously affected by both plant cover and environmental factors. Crop genotypes harbour distinct microbiomes in their different microhabitats and strongly affect microbiome dynamics and assembly. Belowground, soil physicochemical properties including pH, organic matter content, soil texture and nutrient status (Yang et al. 2024) and plant factors such as exudates are the main factors driving the assembly of hostassociated microbiomes (Kudjordjie et al. 2019; Seitz et al. 2024). Compared to the phyllosphere, the soil microbiota is relatively stable, with soil bacteria displaying lower mobility than fungi, which are able to spread through hyphal networks (Hassani et al. 2018; Vos et al. 2013). The phyllosphere microbiome is highly dynamic and is strongly influenced by environmental factors, such as wind, rain, light and temperature, as well as host plant characteristics (Qian et al. 2018; Singh et al. 2018; Szymanski and Miles 2025; Vorholt 2012; Yan et al. 2022). While environmental factors may drive plant microbiomes at macroscale, host phenotypic characteristics have the strongest effects on the phyllosphere microbiome at the microscale (Baltrus 2020; Bell‐Dereske et al. 2023; Szymanski and Miles 2025; Yan et al. 2022). Morella et al. (2020) reported a strong habitat and host genotype‐dependent selection of plant‐associated microbiomes. Yet, detailed empirical studies are still needed to establish microbiome patterns and biogeography at the microscale in diverse cropping systems.

In mixed vegetation types, plant microbiome assembly and their functions are affected by the neighbouring plants, both below‐ and above‐ground (Mészárošová et al. 2024; Meyer et al. 2022). Plant neighbour effects on phyllosphere microbiomes become stronger with increasing biomass of the neighbour plant (Meyer et al. 2022). Different plant genotypes host microbiomes with ecologically distinct functions. Thus, diverse plant neighbours could strongly enhance benefits derived from host‐associated microbiota, including enhancing nutrient acquisition, disease suppression and soil biodiversity (Huss et al. 2022; Liao et al. 2021). For example, maize‐peanut intercropping was reported to alter the composition and diversity of microbial communities in the rhizosphere, including enrichment of beneficial microbes (Zhao et al. 2022). Plant neighbours also modulate pathogen dynamics, for example by affecting the abundance of the fungal pathogen *Rhizoctonia solani* in the focal plant (Ampt et al. 2022). Additionally, neighbouring plants in intercropping systems (two or more crops grown together) or strip intercropping systems (where crops are sown in narrow strips for ecological interaction) (Brennan 2016; Kuerban et al. 2026) may serve as an additional source of microbiome diversity.

We have limited insights into plant microbiome composition, diversity and potential functions in diversified cropping systems. Also, the effects of plant neighbours on the dynamics of the host microbiome are rarely investigated. Hence, empirical studies of plant microbiomes including the effects of plant neighbours on community structuring and diversity in strip‐cropping fields could disclose microbiota signatures and patterns that promote crop health, soil biodiversity and ecosystem stability.

The aim of this study was to investigate the effects of heterospecific neighbour crops on microbial dynamics, including pathogens, below‐ and above‐ground in a strip‐cropping system. Here, we focused on neighbouring spring barley (SB) and faba bean (FB) in a strip‐cropping system. We hypothesised that microbial community structure patterns and dynamics are affected by the neighbouring crop and that this effect is strongest at the edges of strips, where neighbouring crops meet. The specific objectives of our study included (i) characterizing microbial diversity and composition of soil and leaf‐associated microbiomes in gradients from the centre of the strips towards the edge at different time points, (ii) assessing how pathogens are distributed within the crop strips and (iii) examining microbial co‐occurrence network dynamics to infer spatial resilience across the gradients.

## 2 Materials and Methods

### 2.1 Experimental Field Design and Management

A strip‐cropping field was established in 2021 at the Sofiehøj experimental station, Denmark (Coordinates: 54.703353558133315°, 11.445337737133025°), with soils consisting of approximately 19% clay, 50% sand, 2% humus, pH 7.8, 6.4 g kg^−1^ total P, 16.3 g kg^−1^ total K and 6.2 g kg^−1^ total Mg (Table S1). FB, SB, spring or winter wheat, sugar beet, quinoa, rye, pea and oat were grown in the strips and the position of each crop type in the field was rotated annually. In this study, we focused exclusively on the FB and SB strips. These strips were located adjacent to each other and replicated four times, 60 m apart. The strips were 96–180 m long and 6 m wide, with 0.25 m distance between adjacent strips (Figure 1A; Table S1). Climatic data was recorded for the experimental period in 2023 (Table S1). The strip fields were cultivated with spring wheat and sugar beet in April, 2021, and rotated with SB and FB, respectively in March, 2022 and April, 2023 (Table S1). The experimental field layout is outlined in Figure 1A. Sowing was done in rows within each strip at 25 cm distance for SB and FB. Strips were maintained with manual weeding, watering and organic slurry applied to the SB plots prior to seed bed preparation (March), and additionally supplemented with an organic fertiliser, Øgro 10‐3‐1 in 2022 and 2023 (Table S1). Øgro 10‐3‐1 is a 100% recycled organic fertiliser (10% total N, of which approximately 10% is NH_4_^+^‐N) (https://www.oegro.dk/produkter/produktoversigt/oegro‐10‐3‐1/).

**FIGURE 1:**
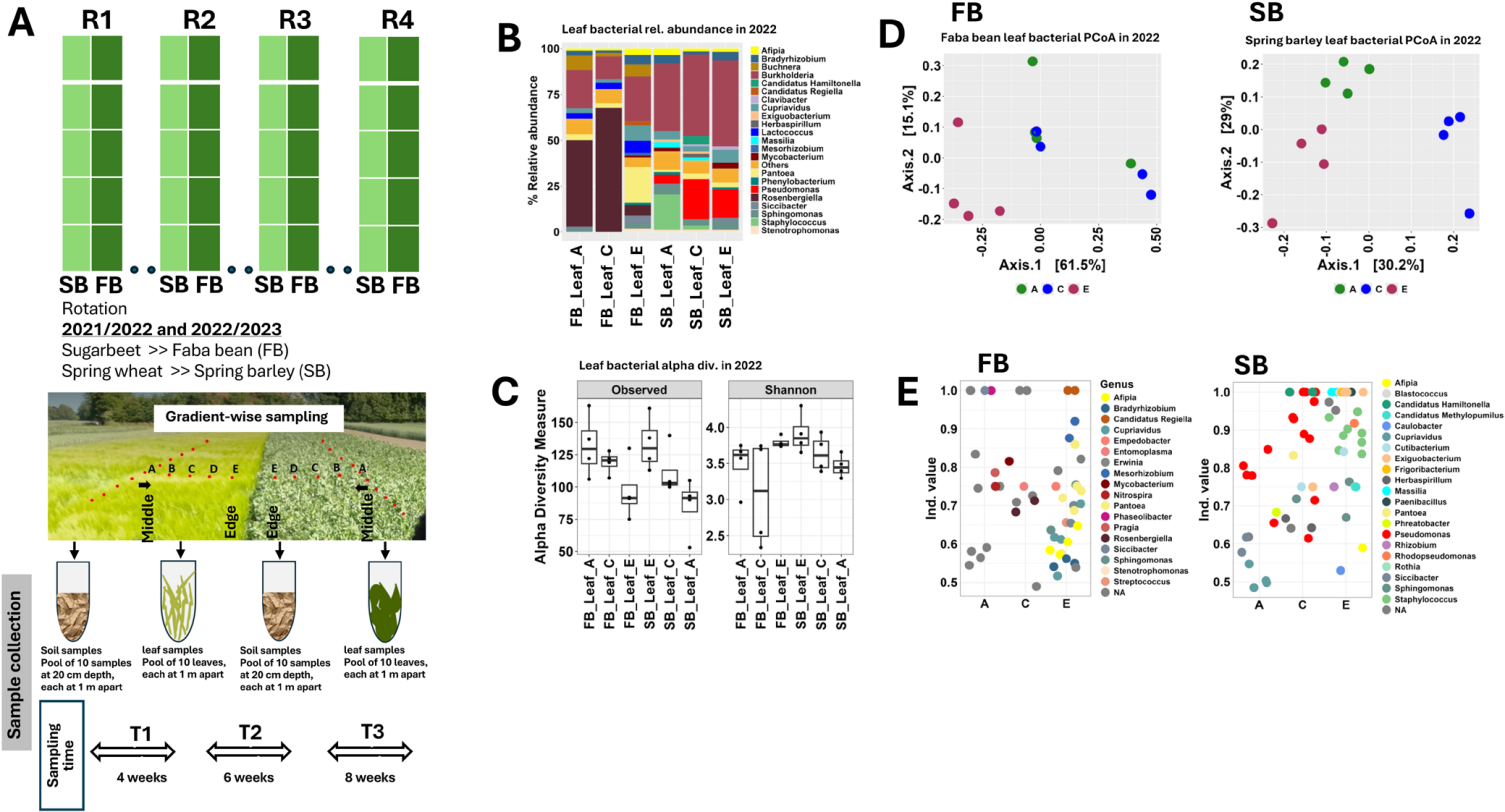
(A) Overview of experimental FB‐SB field design and sampling. Scheme of spring barley (SB) and faba bean (FB) in strip layout including field replication at 60 m distance. Crops cultivated prior to FB and SB in the respective years are also indicated. Snapshot of strip field showing soil and leaf sampling (in red dots) across strip gradient, and time interval of sampling. (B) Bacterial relative abundances, (C) alpha diversity, (D) microbial community structures and (E) indicator species in FB and SB leaf samples collected from centre of strip towards the edge border rows, at sampling time three (T3) in 2022.

### 2.2 Soil and Leaf Sampling

Soil and leaf samples were taken from the FB and SB strips in 2022 (at 2‐week intervals on 18 May, 02 June and 21 June) and in 2023 (at 3‐week intervals on 23 May, 14 June and 06 July) (Figure 1A). Leaf samples were taken in every second row (designated A, C and E) starting from the centre (A) and moving to the edge (E) of the strips, along a 10 m range of rows of FB and SB. One leaf was collected from just below the flag leaf from 10 plants of SB, and similarly a leaf was sampled below the newly formed leaves of 10 SB plants (Figure 1A). Five soil samples (each consisting of 10 pooled sub‐samples sampled along the 10 m range used for leaf sampling) were taken between the rows in a gradient from the centre of the strip and outwards to the edge (A, B, C, D, E). Each soil sub‐sample was collected to a depth of 20 cm using a soil auger (2 cm in diameter).

In 2022, we collected a total of 48 leaf samples (i.e., 2 crop species × 3 gradient positions × 2 sampling times × 4 replicates) and 120 soil samples (i.e., 2 crop species × 5 gradient positions × 3 sampling times × 4 replicates). In 2023, we collected samples at 5 sampling gradient positions each for soils and leaves, thus resulting in a total of 120 leaf samples (i.e., 2 genotypes × 5 gradient positions × 3 sampling times × 4 replicates) and 120 soil samples (i.e., 2 genotypes × 3 gradient positions × 3 sampling times × 4 replicates). The collected samples were placed in plastic bags in cold containers until later storage at −20°C prior to DNA extraction.

### 2.3 DNA Extraction

Leaf and soil samples were homogenised, followed by lyophilization for 72 h and ground using sterile steel beads (5 mm, Qiagen, Hilden, Germany) in a Geno/Grinder2000 at 1500 strokes per min for 3 × 30 s. We used 250 mg of each sample for DNA extraction. Leaf DNA was extracted using the DNeasy Plant Mini kit (Qiagen, Hilden, Germany) while soil DNA was extracted using the PowerLyzer PowerSoil DNA Isolation Kit (Mo Bio Laboratories, Carlsbad, CA), according to the manufacturers´ instructions. DNA concentrations were confirmed using a Qubit dsDNA HS (High Sensitivity) Assay Kit (Invitrogen, Thermo Fisher Scientific, Waltham, MA). DNA samples were stored at −20°C until used for amplicon library preparation.

### 2.4 Library Preparation and Amplicon Sequencing

The bacterial amplicon library was generated by amplifying the 16S rRNA V5–V7 region with 799F/1193R primer pairs (Beckers et al. 2016; Bodenhausen et al. 2013; Chelius and Triplett 2001). The PCR reaction was performed in a 25 μL reaction mix consisting of 12.5 μL Invitrogen Platinum SuperFi PCR master mix (Thermo Fisher Scientific, Waltham, MA), 2 μL of each primer (10 μM stock), 6.5 μL nuclease‐free water and 2 μL of the template DNA. PCR amplification was performed in a GeneAmp PCR System 9700 thermal cycler (Thermo Fisher Scientific, Waltham, MA) using the following conditions: 94.0°C for 3 min, 35 cycles at 94°C for 1 min, 55°C for 1 min, 72°C for 1 min and a final extension step at 72°C for 10 min. The fungal amplicon library was prepared using the fITS7 and ITS4 (Ihrmark et al. 2012; White et al. 1990) fungal primer pair that amplify the internal transcribed spacer 2 (ITS2) region of the fungal rRNA gene (Ihrmark et al. 2012). The amplification was done in a reaction mixture of 25 μL as described for the bacterial amplifications. The thermal cycling conditions for the fungal PCR were 94.0°C for 5 min, 35 cycles at 94°C for 30 s, 57°C for 30 s and a final extension step at 72°C for 30 s. For both bacterial and fungal libraries, we used dual indexing in combination with internal barcodes to enable pooling of samples. The bacterial and fungal forward primers were tagged with varying bases of multiplex identifiers (Kudjordjie et al. 2025, 2019). For indexing, primers including indexing tags were used in a PCR with 10 cycles, with the thermal cycler programs as described above. PCR amplicon sizes of both bacterial and fungal libraries were confirmed on 1.5% agarose gels and expected band sizes excised and extracted using the QIAquick Gel Extraction Kit (Qiagen, Hilden, Germany). The libraries were sequenced on an Illumina MiSeq sequencer with PE300 at Eurofins MWG (Ebersberg, Germany).

### 2.5 Sequence Data Processing and Statistical Analysis

Bacterial and fungal sequences were analysed using the DADA2 (v. 1.12) (Callahan et al. 2016) in the R statistical package (R Core Team, Vienna, Austria). Briefly, the raw reads were quality filtered and trimmed (filterAndTrim—maxN = 0, maxEE = 2, truncQ = 2), followed by error learning (learnErrors), dereplication (derepFastq) and merging of forward and reverse reads (mergePairs) prior to construction of the sequence table (makeSequenceTable) and chimera removal (removeBimeraDenovo). Taxonomic assignment was done using the reference databases SILVA version 128 (Quast et al. 2013) and the UNITE database (Nilsson et al. 2019) train sets (https://benjjneb.github.io/dada2/training.html), for bacterial 16S rRNA and fungal ITS, respectively, using the implementation of the naive Bayesian classifier in the DADA2 R package. Unassigned amplicon sequence variants (ASVs) at kingdom level and ASVs assigned to chloroplast or mitochondrial sequences were excluded from all the datasets.

Statistical analyses and visualizations were performed in R v3.3.0 (R Core Team 2022) using vegan (v2.5.7) (Oksanen et al. 2020), phyloseq (v1.34.0.) (McMurdie et al. 2013) and ggplot2 (v3.3.2) (Wickham 2009) packages. The filtered datasets were normalised and used for visualizing microbial relative abundances in the samples. We examined the significance of differences in the relative abundances of specific pathogenic fungi using a two‐group White's nonparametric *t*‐test in the STAMP software v2.1.3 (Parks et al. 2014; Parks and Beiko 2010), with the Benjamini–Hochberg FDR to control for multiple testing (Benjamini and Hochberg 1994). We assessed alpha diversity (observed richness and Shannon diversity) and statistically significant differences using the non‐parametric Kruskal–Wallis test followed by Bonferroni–Dunn multiple comparison test between groups. The ASV tables were transformed into relative abundances prior to beta diversity analysis. We visualised the Bray–Curtis dissimilarity matrices using unconstrained principal coordinates analysis (PCoA). Following, we performed permutation analysis of variance (PERMANOVA) statistical tests to determine the effects of experimental factors on the community dissimilarity using “adonis” in the vegan package, with 1000 permutations. Pairwise comparisons were performed to assess differences between microbial communities across the sampling gradients using the pairwiseAdonis function (https://github.com/pmartinezarbizu/pairwiseAdonis).

In addition, indicator species analysis (Dufrene and Legendre 1997) was performed to identify microbial ASVs that were significantly associated with specific sampling gradient positions using the indval function in the labdsv package (Roberts 2013). Significant bacterial ASVs (bASVs) and fungal ASVs (fASVs) were defined as those with *p* < 0.01 and an indicator value of at least 0.4.

### 2.6 Correlation Analysis and Microbial Co‐Occurrence Networks

We constructed gradient‐specific microbial co‐occurrence networks as described previously (Kudjordjie et al. 2022), to explore interactions between specific microbial taxa at specific gradient positions in the leaves and soil of FB and SB. Briefly, we combined bacterial and fungal datasets and normalised them using the trimmed mean of M values (TMM) method using the package EdgeR (Robinson et al. 2010). Correlation analysis was performed using ASVs that were present in at least 10 samples with Spearman's rank correlation coefficients > 0.4 (positive correlations) or < −0.4 (negative correlations), and correction using Benjamini–Hochberg FDR. Significant correlations were visualised at the genus level using heat maps. Next, we constructed microbial co‐occurrence networks at the phylum level with ASVs set as nodes and correlations as edges. Network metrics such as transitivity or clustering coefficient (the probability that the adjacent nodes are connected), mean degree (the average number of edges across all nodes in a network), density (fraction of all possible edges actually realised), average path length (APL) (average number of steps which would be required to reach from one node to another in the network) and modularity (measures how well the network is organised into distinct modules) were computed, using the “igraph” package (Csardi and Nepusz 2006). Furthermore, we computed the hub or keystone taxa representing the top 5% of the ASVs with the most correlations in each of the constructed networks. Highly connected ASVs were identified and their relative abundances across were visualised.

To examine whether microbial community resilience differs across the gradients within strips, we assessed the robustness of each co‐occurrence network based on network attack tolerance strategies using the NetSwan package for R [https://cran.r‐project.org/web/packages/NetSwan/index.html]. Each location‐specific network was assessed using four attack to vulnerability strategies involving systematic node removal. These strategies included (i) random node removal, (ii) direct removal, where nodes were removed in descending order of their BNC (betweenness centrality) value (i.e., number of times a node is found on the shortest path between other nodes), (iii) removal of nodes with the highest impact closeness (degree) and (iv) cascading removal, involving the recalculation of BNC values after each node was removed. The effect of each attack metric on network stability was visualised using connectivity loss as a function of fraction of nodes removed.

## 3 Results

### 3.1 Microbiome Composition, Diversity and Indicators in Strip Fields

Bacterial and fungal sequence reads from soil and leaves, with read numbers and sequence read statistics in 2022 and 2023 are summarised in Tables S2 and S3. We removed leaf bacterial reads at the second sampling time (T2) in 2022 due to poor quality.

### 3.2 Microbial Community Structures in 2022

In 2022, leaf bacterial relative abundances were highly distinct across the gradients of both FB and SB strips at T3 (Figure 1B). For example, the bacterial genus *Rosenbergiella* (gradient point A vs. C) and the family Enterobacterales (A and C vs. E) were more abundant in the centre of the strips in FB. In contrast, *Mesorhizobium* and *Pantoea* were more abundant at the edges of the strips (E) (Figures 1B and S1A,B). In the SB leaves, the relative abundance of *Pseudomonas* and *Burkholderia* decreased from the centre towards the edge of strips, while *Staphylococcus* and a significantly abundant taxa *Frondihabitans* had a contrasting trend (Figures 1B and S1C). We observed a significantly decreasing leaf bacterial richness between the centre (A) and edge (E) in SB (Figure 1C, Table S4). Overall leaf bacterial community composition was clearly distinct along the gradients in both FB and SB (Figure 1D) as also confirmed by PERMANOVA (FB: Adonis, *R* ^2^ = 0.48; SB: Adonis, *R* ^2^ = 0.49, *p* < 0.01; Table S5). Pairwise PERMANOVA comparisons revealed significant differences within the gradients (Table S5).

Soil bacterial relative abundances were similar across gradients and time in both FB and SB (Figure S2). Bacterial alpha diversities rarely differed significantly between sampling locations, while beta diversities were generally not significant across gradients in 2022 (Supporting Information: results, Figure S3A,B, Table S6).

Indicator species analysis revealed unique clustering of specific bacterial taxa in leaves at the individual gradient positions within the strips of both FB and SB (Figure 1E). Specifically, *Afipia*, *Pantoea*, *Sphingomonas* and *Bradyrhizobium* were distinct indicators for the edge of the FB strips (Figure 1E). In SB, *Pseudomonas* and *Sphingomonas* strongly associated with the centre of the strips (A, C) while *Staphylococcus* and *Exiguobacterium* were indicators of the strip edges (Figure 1E). Unlike the leaf gradients, indicator bacterial taxa were fewer across the soil gradients of the strips (Figure S3C).

Relative abundances of fungal taxa in leaves varied across the gradients in 2022 (Figure 2A). Most remarkably, in SB, *Puccinia*, containing the pathogen *Puccinia hordei*, was steeply decreasing from the centre towards the edge of the strips at the first two sampling times while this gradient had disappeared at T3 (Figures 2A,B and S4A,B). Although soil fungal relative abundances were similar across the gradients within SB and FB, *Lasionectria* was noticeably enriched in SB compared to FB (Figure S5A,B). There was an overlap in the ASVs occurring in leaves and soils, so we further assessed the fungal pathogen dynamics in the soil and found that *Cladosporium* and *Fusarium* were significantly different between soil samples from the centre and edge locations at T1 and T2 (Figures S6 and S7). Generally, trends in leaf fungal alpha diversity varied at the different time‐points (Figure 2C, Table S7), and distinct fungal communities across the spatial gradients in both FB and SB (Figure 2D), which were confirmed by PERMANOVA and pairwise tests (Table S5). However, soil fungal alpha diversity was rarely distinct across sites (Figure S8A, Table S6) but community composition markedly clustered at specific gradient positions in both FB and SB (Figure S8B). The gradient effect was consistently significant in SB and further confirmed by pairwise comparisons of sampling positions within the gradients (Table S6). Indicator leaf and soil fungal taxa were not distinct across sampling gradients (Figure S9A–F).

**FIGURE 2:**
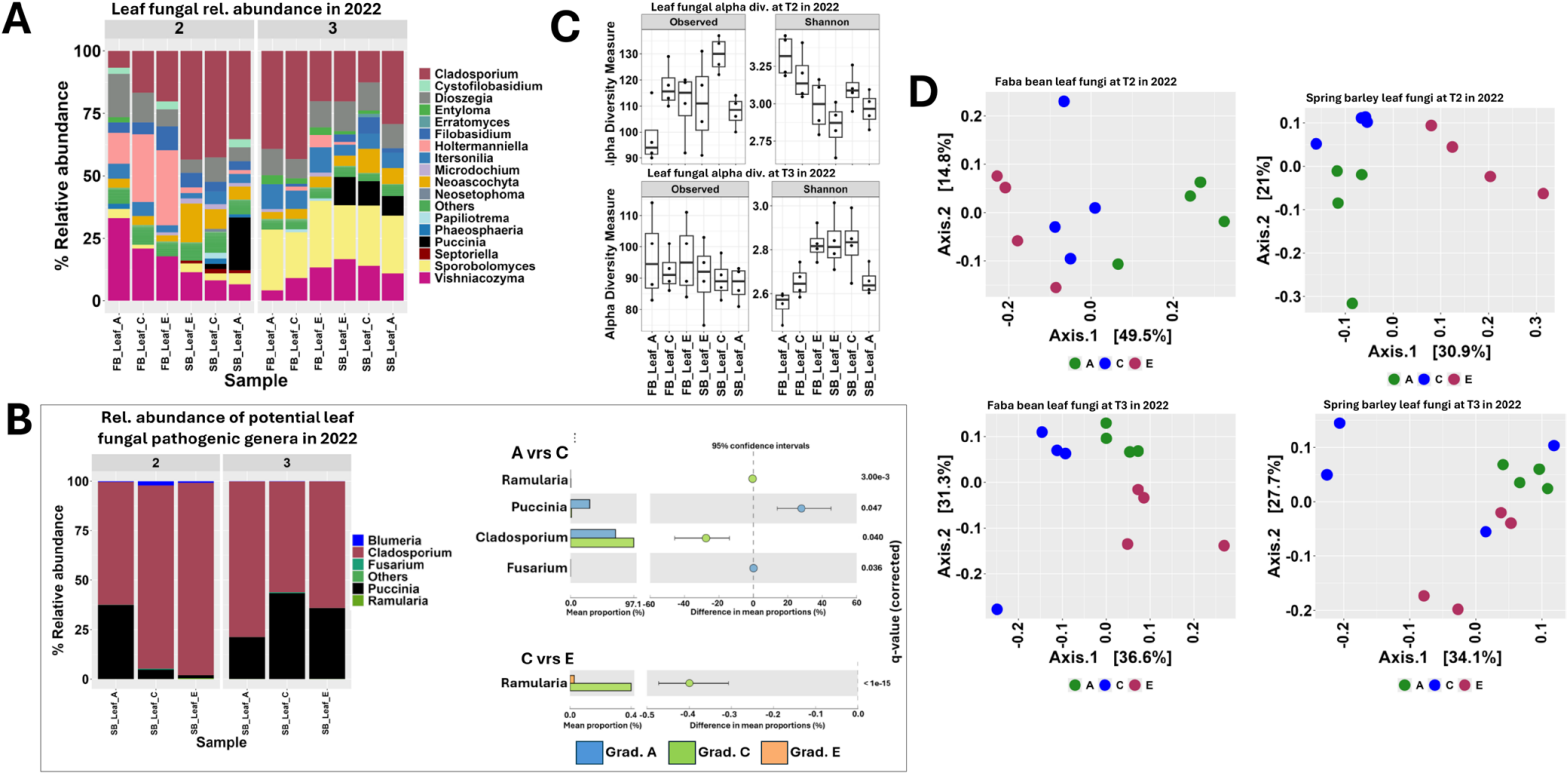
Leaf fungal communities within and across FB and SB strips in 2022. (A) Bacterial relative abundances, (B) relative abundances of potential fungal pathogenic taxa in SB, (C) alpha diversity, (D) microbial community structures in FB and SB leaf samples collected from centre of strip towards the edge rows.

### 3.3 Microbial Community Structures in 2023

We found distinct leaf bacterial relative abundances at different gradient positions, likewise observed shifts in these abundances at the different sampling times in both SB and FB (Figure S10A). Generally, *Burkholderia* dominated the leaf bacterial communities in both SB and FB at all sampling times (Figure S10A). Soil bacterial relative abundances were similar across gradients and time (Figure S10B). In the leaves, the relative abundance of *Puccinia* was highest in the centre of the SB strips, confirming the observations from 2022 (Figure 3A,B). *Cladosporium* was significantly higher at the edges of the strips, while *Blumeria* was significantly enriched in the centres of the strips in SB (Figure 3C). In soil, fungal relative abundances were distinct between the SB and FB strips (Figure S10C), but similar across the gradients in the strips.

**FIGURE 3:**
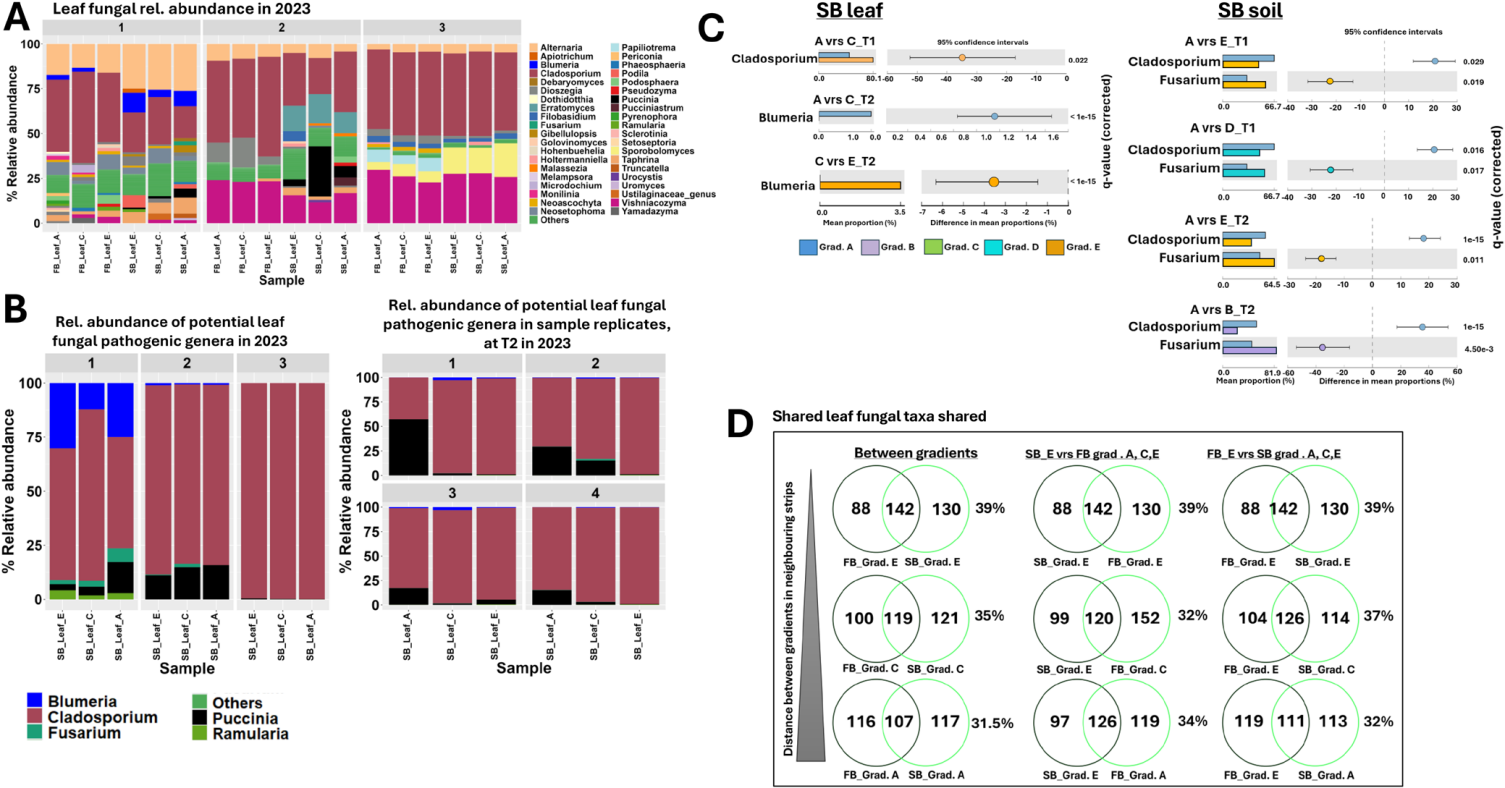
Leaf fungal communities within and across FB and SB strips in 2023. (A) Bacterial relative abundances, (B) relative abundances of potential leaf fungal pathogenic taxa in replicates of SB, (C) relative abundances of potential leaf and soil fungal pathogenic taxa in SB and (D) number of shared leaf fungal taxa at different sampling locations within FB and SB in 2023.

Next, we analysed the proportion of microbial taxa that were present in leaf samples of both FB and SB. Generally, the strip edges (E) had the highest number of shared fungal taxa (39%), followed by position C (35%) and least within the centre positions (A) (31.5%) (Figure 3D). We did not observe notable differences in the proportion of shared bacterial taxa between gradient positions (Figure S11). Microbial alpha diversity was affected, with distinct increasing or decreasing trends across specific sampling locations in the strips (Figures S12–S13; Tables S8 and S9). Leaf microbial communities were markedly distinct across gradients in both FB and SB, with sampling time contributing the strongest variation on microbial communities in leaf and soil (Table 1). Sampling location also had minor but significant effects on FB and SB leaf and soil microbial communities, except in soil bacteria (Figure 4A,B, Table 1, Supporting Information: results). Moreover, the effect of sampling location was highest in fungal communities in both leaves and soil of FB and SB (Figure S14A,B, Table S10). Furthermore, we found indicator species across strip gradients, at different time points in both soil and leaf in SB and FB (Figure S15).

**TABLE 1:** Summary of permutational analysis of variance (PERMANOVA; adonis) performed on Bray–Curtis distance matrices using bacterial and fungal community dissimilarity assessment (2023 dataset) with 1000 permutations.

Dataset	Compartment	Factor	Bacteria (16S) *R* ^2^	Fungi (ITS) *R* ^2^
Faba bean (FB)	Leaves	Sampling location	0.06**	0.07**
		Sampling time	0.56***	0.43***
		Sampling location × Sampling time	0.13***	0.14***
	Soil	Sampling location	0.10**	0.09**
		Sampling time	0.05*	0.18***
		Sampling location × Sampling time	0.18**	0.18*
Spring barley (SB)	Leaves	Sampling location	0.06*	0.06**
		Sampling time	0.62***	0.48***
		Sampling location × Sampling time	0.09***	0.13***
	Soil	Sampling location	ns	0.11***
		Sampling time	0.07*	0.23***
		Sampling location × Sampling time	ns	0.19***

*Note:* Significance of test indicated as ***p < 0.001; **p < 0.01; *p < 0.05. ns denotes not statistically significant and *R* ^2^ is the proportion of variation explained.

**FIGURE 4:**
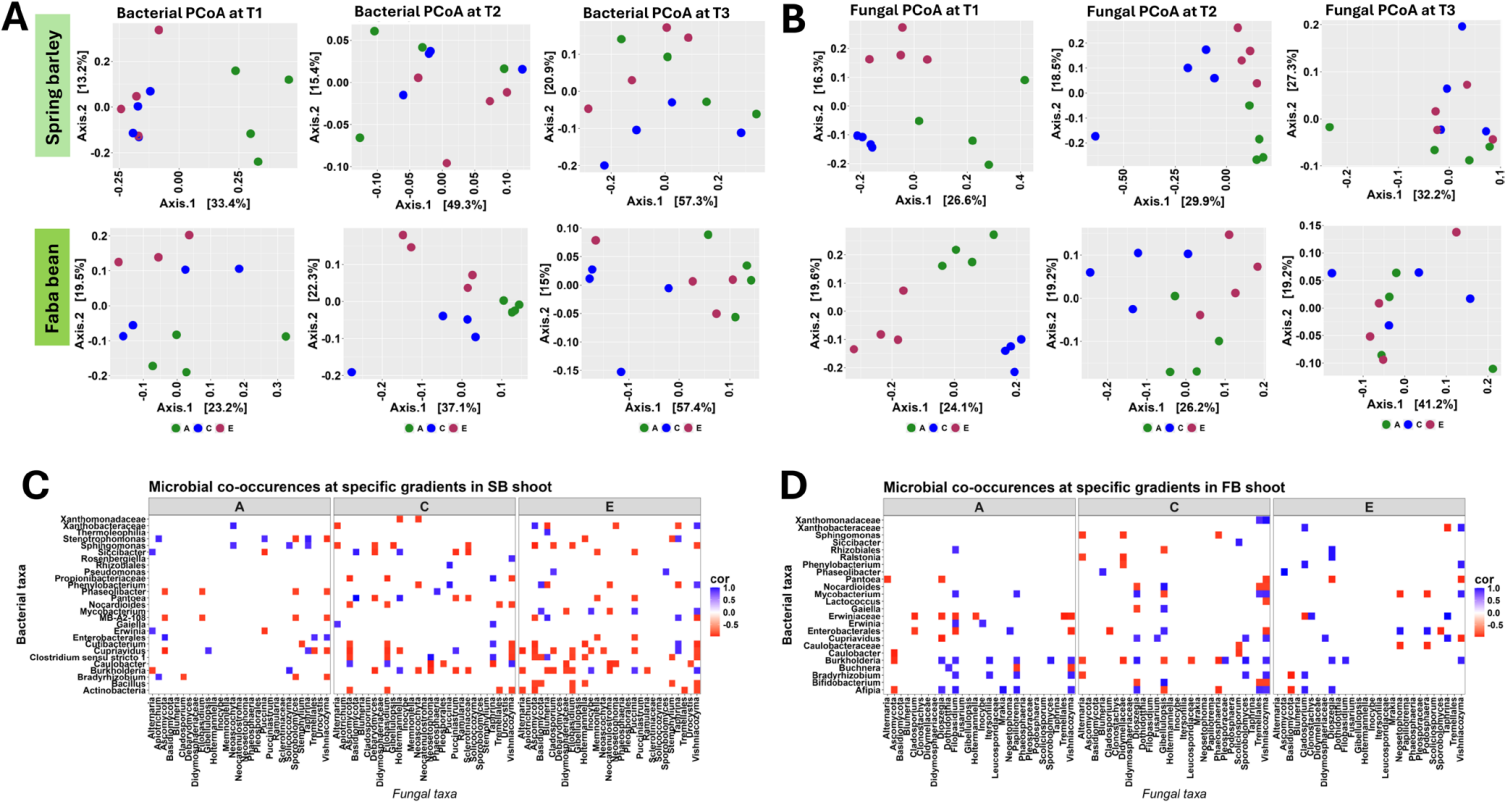
(A) Bacterial and (B) fungal community structures (PCoA visualizations) within and across FB and SB strips at different sampling times, in 2023. Heat map showing significant correlations between bacterial and fungal taxa in (C) SB and (D) FB leaf samples. Bacterial and fungal data from all the time points of 2023 dataset were pooled and used for the analysis. We used ASVs present in at least 10 samples with Spearman's rank correlations coefficients > 0.4 (positive correlations) or < −0.4 (negative correlations), with *p* < 0.05, and correction using Benjamini–Hochberg FDR. Positive and negative correlations are indicated by blue and red colours, respectively.

### 3.4 Gradient‐Specific Microbial Co‐Occurrence Networks in 2023

Gradient‐specific correlation analysis identified positive and negative co‐occurrences among microbial taxa in leaf and soil samples of FB and SB (Figures 4C,D and S16A,B). A higher number of fungal‐bacterial correlations were detected in the leaves of SB compared to FB. In SB, the strip edges had the highest and most negative correlations with *Pantoea* and Enterobacterales correlating negatively with *Puccinia* (Figure 4C). *Burkholderia* correlated negatively with *Alternaria* in the centre (A) and edge (E). Similarly, in FB leaves, *Pantoea* correlated negatively with *Alternaria* in the centre (A), and so did *Sphingomonas*, *Ralstonia*, *Burkholderia* and *Bradyrhizobium* in position C (Figure 4D). In soil, a higher number of negatively correlating taxa was found in centre gradients than the edge in FB, including *Fusarium* correlating negatively with *Bacillus*, *Nitrospira*, *Nocardioides* and *Variovorax* (Figure S16B).

Microbial co‐occurrence networks revealed distinct patterns of bacterial and fungal associations in the different sampling points in FB and SB (Figure 5A,B). Network node and edge numbers (both positive and negative) inferred from each network and additional network properties are summarised in the Table S11. Overall, leaf microbial networks were denser in FB than in SB. The SB leaf networks were the most distinct with highly sparse structures at the centre of the strip to more dense and highly connected networks towards the edge (Figure 5A). Network metrics including mean degree (Mn) and APL corroborated the differences in co‐occurrence networks across gradients in SB. Specifically, the Mn of SB leaf networks were 7.1, 14.6 and 13.3 in positions A, C and E, respectively. Similarly, the APL was highest in networks from the centre of the strip and declined towards the edge (Figure 5A). FB leaf networks also revealed subtle differences in network properties with modularity being highest in the edges of both leaf and soil (Figure 5B). Furthermore, soil gradient specific networks were distinct between FB and SB (Figure S17A,B). We identified distinct hub taxa with varied connections and relative abundances in networks from different positions along the gradient (Figure 5). The *Vishniacozyma* (*V. victoriae*) and *Taphrina* were the dominating hub taxa with high relative abundance in both FB and SB networks. *V. dimennae* and V. *victoriae* were highly abundant hub taxa in the edges, while *Taphrina*, *Sporobolomyces* ( *S. roseus* ) and *Neosetophoma* dominated the centre of SB. In FB, *Burkholderia* and *Papiliotrema* ( *P. flavescens* ) dominated the centre, while *Cladosporium* was the most abundant hub taxa in the edges of FB.

**FIGURE 5:**
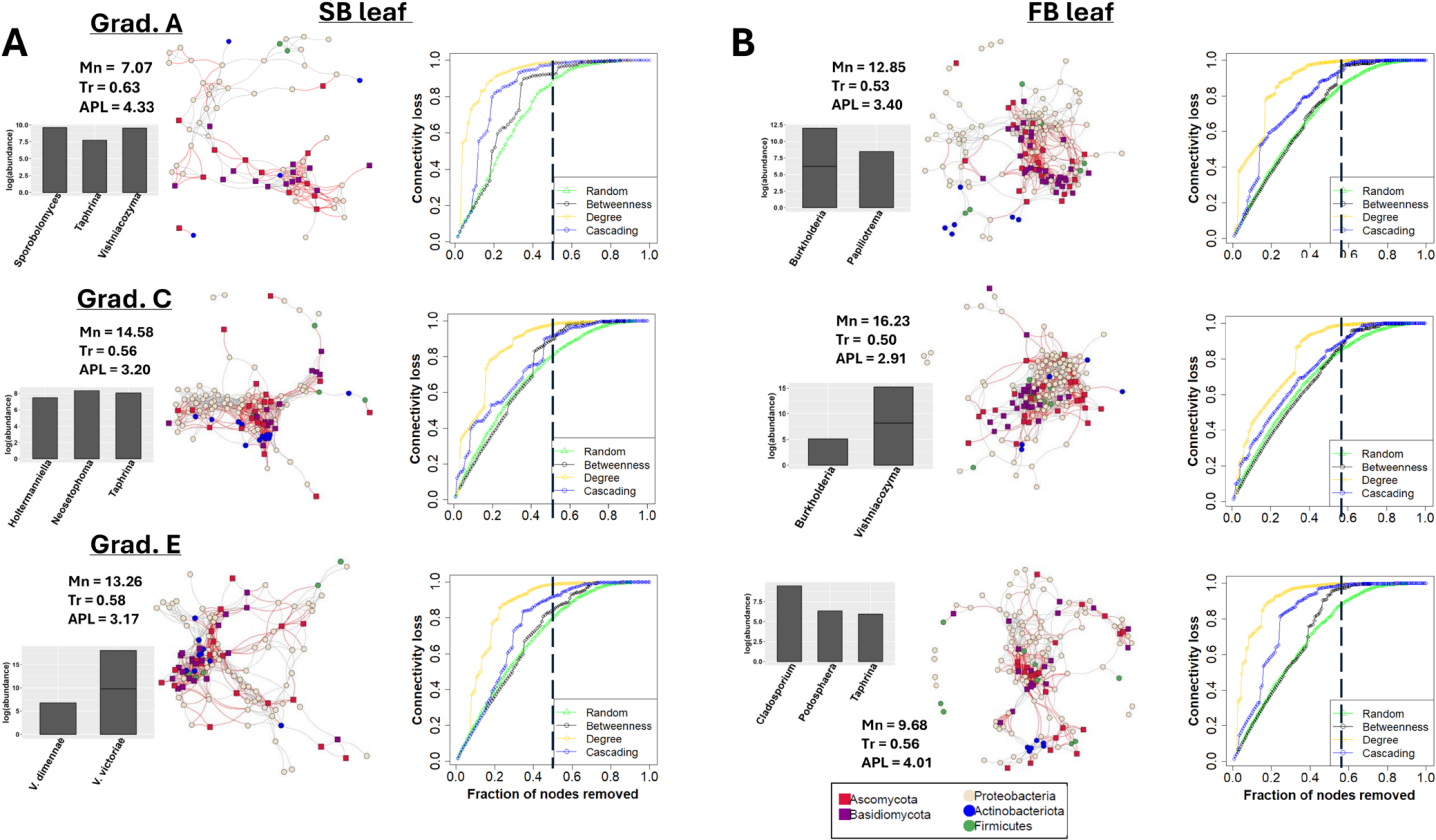
Leaf microbial co‐occurrence networks computed for the different sampling locations (centre to edge) in (A) SB and (B) FB. The grey and red edges represent positive and negative correlations, respectively. Network tolerance to attack of the respective site‐specific networks using change in connectivity as a function of the fraction of removed nodes. The dashed lines show the maximum fraction of nodes to be removed for 100% connectivity loss (highest break‐down of network) are shown. The four sustained attack strategies to test tolerance included random removal, direct removal of nodes with highest betweenness centrality, target on nodes with the highest impact closeness (degree), and a combination of random and targeted on betweenness (cascading). Additionally, relative abundance of hub taxa identified (including the numbers of ASVs assigned to phyla) for each site‐specific network are shown.

We tested the robustness of the networks by assessing their tolerance using four node sustained attack strategies, including random removal, direct removal of nodes with the highest betweenness centrality, targeted removal of nodes with the highest impact closeness (degree) and an approach involving a combination of random and targeted on betweenness (cascading). Among the tested strategies, random removal inflicted least connectivity loss, whereas degree and cascading attacks caused the lowest tolerance in both leaf and soil networks (Figures 5A,B and S17A,B). SB leaf gradient specific networks exhibited a pronounced breakdown vulnerability, which was higher in the centre network compared with edge networks when assessing breakdown using all the attack strategies, except degree attack (Figure 5A). Degree nodes had the highest vulnerability to attack, as removal of 50% nodes generally resulted in a 100% breakdown. For the FB leaf gradient networks, a higher number of node removal (57%) was required to totally disrupt the networks when using degree (for all networks), and for cascading and betweenness of locations in the A and E networks (Figure 5B). Although subtle differences were observed between soil gradient specific networks, attack strategies did not reveal clear differences (Figure S17A,B).

## 4 Discussion

Mixed cropping systems promote high microbial biodiversity and have been suggested to enhance crop growth and disease suppression (Jiang et al. 2024; Trinchera et al. 2022; Wang et al. 2021; Xiao et al. 2023), and plant associated microbiomes have been widely suggested as a “green solution” for sustainable crop production (Diaz‐Troya and Huertas 2024). Yet, we still have limited understanding of the effects mixed cropping systems such as strip‐cropping have on microbiome composition, diversity and dynamics at the field scale. In the present study, we examined how the structure and composition of leaf and soil microbiomes of FB and SB varied from the centre towards the edge of the strips, where the two crops met. Different plant species assemble distinct microbiomes depending on their taxonomic relationship, due to their unique genotypic and phenotypic traits including height, canopy and leaf type, which significantly affect the host microbiome dynamics (Capparotto et al. 2026; Finck et al. 2026; Negre Rodríguez et al. 2025; Zhang et al. 2026). Using different crop species leverages on the ability of distinct crops to assemble diverse microbiomes that have the functional potential to promote plant growth and resilience towards biotic and abiotic stresses. Thus, FB as a dicot and SB as a monocot were expected to assemble different microbiomes, which could potentially affect plant development in the respective strips. We expected that microbiome assembly would be affected by the presence of the adjacent crop, but to what extent the proximity to the adjacent crop would affect microbiome assembly above‐ and below‐ground was less predictable.

### 4.1 Microbial Communities Are Dynamic and Differ Across Strip Crop Spatial Gradients

We found distinct microbial communities in the spatial gradients within the SB and FB strips, thus confirming our hypothesis of neighbour‐effects on the host‐associated microbiomes in the strips. We found that leaf communities were more divergent between SB and FB at the early stages compared to the later stages, indicative of stronger effects of species at early growth stages (Almario et al. 2022). Soil fungal communities were more distinct at later stages, an effect that was not observed in the bacterial communities, suggesting a stronger effect of the host in structuring fungal communities. Both sampling time and gradient position significantly affected the microbial community structures in leaves and soils of FB and SB, except for the soil bacterial communities in SB that did not vary with gradient position. Sampling time was contributing the largest effects on the microbial communities, suggesting effects of host physiological changes and temporal changes of dispersal patterns, especially aboveground (Edwards et al. 2018; Micallef and Colón‐Carmona 2013; Quiza et al. 2023).

Our data clearly revealed distinct leaf microbiota in SB compared to FB, further corroborating the effects of individual host phenotypic traits on microbial community assembly (Yan et al. 2022). While limited work exists comparing microbiome assembly in leaves of monocot and dicots, reports show that broadleaved woody plants hosted higher proportions of N‐fixing bacteria than monocots (Labouyrie et al. 2023). Leaf type, surface microtopography and canopy cover determine the surface area for microbial colonization and affect micro‐environmental factors (temperature, relative humidity, wind) differentially affecting microbial communities (Yan et al. 2022). Also, the consistently more dynamic leaf microbiomes compared to the soil microbiomes are not surprising as highly perturbing weather factors strongly alter the phyllosphere microbiome (Brandl et al. 2023).

### 4.2 Abundance of Fungal Pathogens Varied Across Gradients

Strip‐cropping systems have been suggested as a sustainable way of reducing pathogen pressures. We found that the relative abundance of plant fungal pathogenic genera on SB leaves, specifically *Puccina* was consistently higher in the centres than at the edges of strips, particularly at earlier sampling times. These results were consistent in the individually replicated samples across the field, thus providing strong evidence of *Puccinia* development in the centre of the strips. The genus *Puccinia* contains *P. hordei*, which is causing a destructive leaf rust disease of cereals globally (Singh et al. 2011). Other pathogenic genera, including *Blumeria* (causing powdery mildew), *Ramularia* (causing leaf spot) and *Cladosporium* (causing leaf spot, scab and blights), were also found to be differentially abundant across gradients in both 2022 and 2023. Similarly, profiling of pathogens in soil revealed consistent enrichment of *Cladosporium* in the centre location, while *Fusarium* was enriched at the edge. These findings suggest differing tendencies of fungal pathogenic attack at specific positions within strip fields, as previously reported (Dentika et al. 2022). Whether the microbiota sourced from neighbouring plants (either conspecific or heterospecific) affects the spread of pathogens would require further studies. However, there is evidence that root‐exuded metabolites of neighbour plants regulate plant susceptibility to soil pathogens in intraspecific mixtures (Hama et al. 2025; Pélissier et al. 2021). Similarly, pathogen suppressive volatiles from neighbour plants could result in reduced leaf or root pathogen establishment in a strip‐cropping system (Trivedi et al. 2020; Zhou et al. 2024). Repeated rotation with crops secreting bioactive root exudates could induce soil suppressiveness against microbial pathogens. Moreover, it is interesting to note that pathogenic lifestyles may be plant species dependent, as, for example, a pathogen of dicots could eventually act as a biocontrol agent in monocots (Tian et al. 2020).

We identified indicator microbial taxa associated with the centres or edges of strips. For instance, at T3 in 2022, the bacterial indicators *Pantoea*, *Sphingomonas* and *Bradyrhizobium* were associated with leaves in edges of the FB strips. In the SB strips, *Pseudomonas* and *Sphingomonas* were associated with the centres, while *Staphylococcus* and *Exiguobacterium* were main indicators of the edges in the strips of SB. Several of these taxa have been studied for their biocontrol traits (Finkel et al. 2017). For example, *Pantoea agglomerans* has a strong antagonistic effect against a wide range of pathogens in many plant species (Duchateau et al. 2024).

### 4.3 Interspecific Neighbouring Effects Are Gradient‐Dependent Across Adjacent Crop Strips

Current knowledge of plant effects on the associated microbiomes is still largely based on the intricate interactions between individual plant species and the microbiota, with limited consideration of potential neighbour‐effects on microbiome assembly. We found a higher number of shared fungal genera in leaves from the edges of strips that are close to the neighbouring strips, thus demonstrating an interactive effect of neighbouring crops on the leaf mycobiome. It has been shown that plant neighbouring effects become stronger over time with increasing microbiota dispersal from neighbouring plants as plant biomass increases (Meyer et al. 2022). This neighbouring effect might eventually decrease host filtering effects on microbial communities over time (Meyer et al. 2022). We speculate that the shared microbial taxa emanating from the adjacent neighbours could be affecting overall host performance. For instance, beneficial microbial species dispersed from the neighbouring plant could be antagonising pathogenic microbes that would likely attack a specific focal plant. Further experimental evidence is needed to assess how the newly arrived microbiota from neighbouring crops affects host‐microbial pathogen interactions in strip‐cropping systems.

### 4.4 Distinct Gradient‐Specific Microbial Interactions and Co‐Occurrence Networks Within Strips

Although correlation‐based analyses do not demonstrate causes and effects, it enables us to discern plausible ecological relationships between microbial species, and between microbes and the environment (Liang et al. 2018). Thus, we further examined whether microbial co‐occurrence patterns vary between gradient positions using correlation‐based analysis and network visualization. Positive and negative correlations suggested potential positive and antagonistic interactions, respectively, within the host‐associated microbiota (Hassani et al. 2018). The varying microbial correlations associated with specific gradient positions in the strips supported localised microbial interactions that could affect microbiome‐modulating effects on plant functions. We observed that *Siccibacter* and *Erwinia* correlated negatively with *Puccinia* in the centre, while *Pantoea* and *Cupriavidus* negatively correlated with *Puccinia* in the edges of SB strips. *Erwinia* species have been shown to suppress *Puccinia recondita* (Kempf and Wolf 1989) causing wheat leaf rust (Krępski et al. 2024), and *Siccibacter colletis* was reported to promote wheat growth via enhancing phosphate and potassium uptake (Kumar et al. 2024). Similarly, *Pantoea* species are notably known for their biocontrol activities against several pathogens (Duchateau et al. 2024). Thus, these different microorganisms could potentially be contributing to the control of pathogens at different positions along the gradient from centre to edge of the strips.

As expected, inter‐phylum co‐occurrence networks exhibited a more dissimilar configuration in the leaf than in the soil gradients, primarily due to more dynamic patterns of the phyllosphere microbiota. Also, leaf networks varied more across the gradient in SB compared to FB, thus corroborating distinct community dynamics in grasses and broad leaf plants. In SB, leaf networks showed greater microbial community connectivity and robustness to disturbances at the edges, indicative of diverse crops assembling more robust microbiomes. Network metrics also reveal insights into the microbial community dynamics during external perturbations. For instance, the transitivity and low APL characterizing the SB edge network indicate a more connected and robust network (Kudjordjie et al. 2024) compared with the centre networks. The robust SB edge network could suggest stronger microbial competition (Wagg et al. 2019), which could further decrease pathogen establishment. While FB leaf networks were quite similar, the high modularity characterizing edge networks suggests higher resilience of these communities (Gao et al. 2021; Lurgi et al. 2014). However, detailed studies are needed to validate the biological relevance of these interactions at individual gradient positions.

We found hub taxa with different occurrences along the strip gradients indicative of the role of specific microbial taxa in community structuring. Keystone taxa are known to modulate several functions, which can include regulating the invasion of fungal pathogens (Trivedi et al. 2017). *Sporobolomyces roseus* , a dominant hub taxon at the centre position of SB, has been reported to be antagonistic against *Botrytis cinerea* (Fenta et al. 2020) and *Cochliobolus sativus* (Bashi and Fokkema 1977) (Zhong et al. 2015) via nutrient competition. Moreover, *Vishniacozyma victoriae*, a hub taxon in both SB and FB networks, is suppressive against *B. cinerea* (Gorordo et al. 2022; Nian et al. 2023), and *Penicillium expansum*, and *Cladosporium* sp. (Gorordo et al. 2022). Also, *Papiliotrema flavescens*, a dominant hub taxon at the edge of FB strips, has notable plant growth‐promoting properties including induction of systemic resistance against *B. cinerea* in *Arabidopsis* (Liu et al. 2024). Additionally, *Burkholderia* is known for biocontrol traits against a wide range of plant pathogens (Compant et al. 2008). Altogether, these results demonstrate that keystone species spatially distributed within strips could be driving microscale co‐occurrence community interactions.

### 4.5 Leaf Co‐Occurrence Networks Are Most Robust at the Edges of SB Strips

Network tolerance to disturbance confirms the higher robustness of the edge than centre networks in SB leaves. Networks with higher robustness are able to resist rapid collapse caused by disturbance, for example pathogen attack (Kudjordjie et al. 2024), and thus have greater stability (Kajihara and Hynson 2024). These results suggest that the strip edges are more resilient, while the centres with conspecific plants are expected to be more vulnerable to pathogen attack. Interestingly, these results support the higher number of negative correlations detected at the edges; likewise, a higher neighbour effect was revealed in the number of shared microbial taxa. However, the larger gradient‐specific variation in the SB compared with the FB suggests that community resilience within strips is host‐dependent. Moreover, since networks are dynamic due to the continuous influence of neighbour host and environmental factors (Kajihara and Hynson 2024), the observed site‐specific microbial co‐occurrences would change across time and would correspondingly alter resilience patterns within strip‐crop fields.

## 5 Conclusions

The present work investigated the dynamics and site‐specific assembly of soil and leaf microbiomes in a two‐year rotational FB and SB strip field using Illumina amplicon sequencing. We found distinct microbial composition and diversity characterizing both above and belowground sampling positions within and between SB and FB strips. Relative abundances of fungal pathogens, such as *Puccinia*, *Blumeria* and *Cladosporium*, varied across strip gradients; specifically, *Puccinia* was consistently more abundant in strip centres in SB. Moreover, the effects of neighbouring strips on microbial richness were highest close to the edges. Additionally, we revealed distinct site‐specific microbial interactions and co‐occurrence networks within the strips. Inter‐phylum leaf co‐occurrence networks exhibited more dissimilar network configurations compared with the soil networks in SB, primarily due to more dynamic patterns of the leaf microbiota. Network tolerance to disturbance confirmed more resilient networks at the edges of the SB strips. The distinct microbial co‐occurrences exemplified by network structure, topology and hub taxa could explain varying community resilience that characterises strips with different crops and sites within strips. Altogether, we show that (i) strip‐cropping has a much higher impact on spatial variability of leaf microbiomes than soil microbiomes, (ii) neighbouring crop species harbour host‐adapted microbiomes, but with increasing proximity to the neighbouring crop, their microbiomes share more microbial taxa, (iii) strip‐cropping may reduce pathogens in leaves, depending on crop and pathogen species. These findings will facilitate dissection of crop field‐level microbiome assembly and further, the generation of mixed‐host mechanistic hypotheses of microbiome dynamics positively promoting sustainable cropping systems.

## Author Contributions

**Enoch Narh Kudjordjie:** conceptualization, methodology, software, data curation, investigation, validation, formal analysis, visualization, writing – original draft, writing – review and editing. **Mette Vestergård:** conceptualization, methodology, investigation, validation, supervision, writing – review and editing, visualization, data curation. **Mesfin Tsegaye Gebremikael:** conceptualization, methodology, writing – review and editing, data curation. **Otto Nielsen:** conceptualization, methodology, investigation, validation, supervision, funding acquisition, data curation, project administration, resources, writing – review and editing. **Mogens Nicolaisen:** conceptualization, methodology, investigation, validation, supervision, project administration, resources, writing – review and editing, funding acquisition, visualization, data curation.

## Funding

This work was supported by GUDP Organic RDD 6 project, number 34009‐20‐1703.

## Ethics Statement

The authors have nothing to report.

## Consent

The authors have nothing to report.

## Conflicts of Interest

The authors declare no conflicts of interest.

## Supporting information


**Data S1:** Supporting Information.
**Table S2:** Sequence statistics including mean, median and range of reads per compartment for fungal and bacterial libraries at different days post‐inoculation (DPI) in 2022.
**Table S3:** Sequence statistics including mean, median and range of reads per compartment for fungal and bacterial libraries at different days post‐inoculation (DPI) in 2023.
**Table S4:** Leaf bacterial alpha diversity pair‐wise comparison (using Wilcoxon rank sum test) between strip gradients (from middle of strip towards the edge border rows [A–E]) of faba bean (FB) and spring barley (SB) samples at different sampling time T3 in 2022.
**Table S5:** Summary of permutational analysis of variance (PERMANOVA) and Pairwise Adonis at each sampling time in 2022 using the “adonis” test on Bray–Curtis distance matrices using bacterial and fungal community dissimilarity assessment using 1000 permutations.
**Table S6:** Soil bacterial and fungal alpha diversity pair‐wise comparison (using Wilcoxon rank sum test) between strip gradients (from middle of strip towards the edge border rows [A–E]) of faba bean (FB) and spring barley (SB) samples at different sampling time T2 and T3 in 2022.
**Table S7:** Fungal leaf alpha diversity pair‐wise comparison (using Wilcoxon rank‐sum test) between strip gradients (from middle of strip towards the edge border rows [A–E]) of faba bean (FB) and spring barley (SB) samples at different sampling time T2 and T3 in 2022.
**Table S8:** Leaf alpha diversity pair wise comparison (using Wilcoxon‐rank sum test) between strip gradients (from middle of strip towards the edge border rows [A–E]) of faba bean (FB) and spring barley (SB) samples at different sampling times in 2023.
**Table S9:** Soil alpha diversity pair wise comparison (using Wilcoxon‐rank sum test) between strip gradients (from middle of strip towards the edge border rows [A–E]) of faba bean (FB) and spring barley (SB) samples at different sampling times in 2023.
**Table S10:** Summary of permutational analysis of variance (PERMANOVA) and Pairwise Adonis at each sampling time in 2023 using the “adonis” test on Bray–Curtis distance matrices using bacterial and fungal community dissimilarity assessment using 1000 permutations.
**Figure S1:** Leaf bacterial genera with significantly different relative abundances between sampling locations (A) A and E and (B) C and E in FB and (C) C and E in SB at T2 in 2022. Analysis was performed with the STAMP software with White's nonparametric *t*‐test, followed by Benjamini–Hochberg correction (FDR) with q‐value < 0.05. The statistical test used was two‐sided.
**Figure S2:** Soil bacterial relative abundance in faba bean (FB) and spring barley (SB) samples across strip gradients (from centre of strip towards the edge border rows [A–E]) at different sampling times in 2022.
**Figure S3:** (A) Soil bacterial alpha diversity in faba bean (FB) and spring barley (SB) samples across strip gradients (from centre of strip towards the edge border rows [A–E]) at different sampling times in 2022. (B) Principal coordinate analysis (PCoA) plots of soil bacterial communities at different sampling sites in FB and SB strips at different time points in 2022. (C) Soil indicator bacterial species characterizing different gradient positions in FB and SB spring fields in 2022.
**Figure S4:** Relative abundance of potential SB leaf fungal pathogenic genera in replicates at sampling times (A) T2 and (B) T3 in 2022.
**Figure S5:** Relative abundance of soil fungi at sampling time (A) 1, 2 and (B) sampling time 3 in 2022.
**Figure S6:** Relative abundance of potential soil fungal pathogens in SB sample replicates for the different sampling time points in 2022.
**Figure S7:** Differences in the abundances of potential fungal pathogenic genera in soil between gradient positions at T1 and T2 in SB. Analysis was performed with the STAMP software with White's nonparametric *t*‐test, followed by Benjamini–Hochberg correction (FDR) with *q*‐value < 0.05. The statistical test used was two‐sided.
**Figure S8:** (A) Soil fungal alpha diversity in faba bean (FB) and spring barley (SB) samples across strip gradients (from centre of strip towards the edge border rows [A–E]) at different sampling times in 2022. (B) Principal coordinate analysis (PCoA) plots of soil fungal communities at different sampling sites in FB and SB strips at different time points in 2022.
**Figure S9:** Indicator leaf (A,B) and soil (C–F) fungal species characterizing different sampling locations in FB and SB spring fields in 2022. CD using T1, T2 and EF using T3 datasets, respectively.
**Figure S10:** (A) Leaf bacterial, (B) soil bacterial and (C) soil fungal relative abundances in faba bean (FB) and spring barley (SB) samples across strip gradients (from centre of strip towards the edge border rows [A–E]) at different sampling times in 2023.
**Figure S11:** Number of shared leaf bacterial taxa at different sampling locations within FB and SB in 2023.
**Figure S12:** (A) Leaf and soil bacterial alpha diversity in faba bean (FB) and spring barley (SB) samples across strip gradients (from centre of strip towards the edge border rows [A–E]) at different sampling times in 2023.
**Figure S13:** (A) Leaf and soil fungal alpha diversity in faba bean (FB) and spring barley (SB) samples across strip gradients (from centre of strip towards the edge border rows [A–E]) at different sampling times in 2023.
**Figure S14:** Principal coordinate analysis (PCoA) plots of soil (A) bacterial and (B) fungal communities at different sampling sites in FB and SB strips at different time points in 2023.
**Figure S15:** Indicator bacterial and fungal species characterizing different leaf and soils at different sampling locations in FB and SB spring fields in 2023.
**Figure S16:** Heat map showing significant correlations between bacterial and fungal taxa in (A) SB and (B) FB soil samples. Bacterial and fungal data from all the time points were pooled and used for the analysis. We used ASVs present in at least 10 samples with Spearman's rank correlations coefficients > 0.4 (positive correlations) or < −0.4 (negative correlations), with *p* < 0.05, and correction using Benjamini–Hochberg FDR. Positive and negative correlations are indicated by blue and red colours, respectively.
**Figure S17:** Soil microbial co‐occurrence networks computed for the different sampling locations (centre to edge sites) in (A) SB and (B) FB samples. The grey and red edges represent positive and negative correlations, respectively. Network tolerance to attack of the respective site‐specific networks using change in connectivity as a function of the fraction of removed nodes. Dashed lines show the maximum fraction of nodes to be removed for 100% connectivity loss (highest breakdown of network). The four sustained attack strategies to test tolerance included random removal, direct removal of nodes with highest betweenness centrality, target on nodes with the highest impact closeness (degree) and a combination of random and targeted on betweenness (cascading). Additionally, relative abundances of hub taxa identified (including the numbers of ASVs assigned to phyla) for each site‐specific networks are shown.

**Table S1:** Field management, soil properties and climate data.

**Table S11:** Summary of network properties including edges, nodes and hub species.

## Data Availability

The MiSeq paired‐end reads obtained from the bacterial 16S rRNA gene (V3–V4) and fungal ITS2 gene regions have been deposited in NCBI SRA repository, under BioProject ID PRJNA1345709.
