# Supplementary material for: Neighbour Effects and Dynamics of Plant‐Associated Microbiomes in a Strip‐Crop System
Source: Environ Microbiol Rep. 2026 Oct 1;18(5):e70423. doi: 10.1111/1758-2229.70423 (PMC13631382; doi:10.1111/1758-2229.70423)
Supplement: Supplementary file 1 [file EMI4-18-e70423-s001.pdf]

# Supplementary file
# Neighbour effects and dynamics of plant-associated microbiomes in a strip-crop system

Enoch Narh Kudjordjie[1]*, Mette Vestergård[1], Mesfin Tsegaye Gebremikael[2], Otto Nielsen[3], Mogens Nicolaisen[1]*

[1]Department of Agroecology, Faculty of Technical Sciences, Aarhus University, 4200 Slagelse, Denmark

[2]Department of Food Science - Plant, Food & Sustainability, Department of Food Science - Food Technology. Agro Food Park 48, 8200 Aarhus N, Denmark

[3]Nordic Beet Research (NBR), Sofiehøj, Højbygårdvej 14, 4960 Holeby, Denmark

*Correspondence: Enoch Narh Kudjordjie (enochnarh@agro.au.dk), Mogens Nicolaisen (mn@agro.au.dk)

# Supplementary results

**Beta diversity in 2022**

Soil bacterial PCoA plots showed that bacterial community composition was distinct for specific sampling locations in both FB and SB, but this was time dependent (**Supplementary Figure S3B**). PERMANOVA showed significant effects of gradients on bacterial communities only at the earliest sampling time in FB (sampling time 1; **Adonis, $R^2$ = 0.29, $P< 0.01$**) and in both earlier sampling times in SB (sampling time 1; **Adonis, $R^2$ = 0.32, $P< 0.001$;** sampling time 2; **Adonis, $R^2$ = 0.26, $P< 0.05$**) (**Supplementary Table S5**). Pairwise PERMANOVA comparisons confirm specific location that are significantly different in both between the compared locations (**Supplementary Table S5**).

**Beta diversity in 2023**

PERMANOVA confirmed significant proportion of variation explained by both sampling location and time on leaf and soil microbial community structures of FB and SB. The effect of sampling time was strongest on both crop's leaf bacterial (FB; **Adonis, $R^2$ = 0.56,** SB; **Adonis, $R^2$ = 0.62, $P<0.001$, Table 1)**, and fungal (FB; **Adonis, $R^2$ = 0.43,** SB; **Adonis, $R^2$ = 0.48, $P<0.001$, Table 1)** communities. However, the interactive effects of both factors were also stronger on leaf microbial communities (FB; **Adonis, $R^2$ = 0.13,** SB; **Adonis, $R^2$ = 0.09, $P<0.001$, Table 1)**, and fungal (FB; **Adonis, $R^2$ = 0.14,** SB; **Adonis, $R^2$ = 0.13, $P<0.001$, Table 1)** than sampling location alone**.** In soil, sampling time affected microbial communities of both FB and SB, but these effects were stronger on fungi than bacterial communities. Also, sampling location contributed significant variation on microbial communities, except on the bacterial communities in SB (**Table 1)**. Similarly, the interactive effects of these factors strongly altered microbial communities in FB and on fungal communities in SB (**Table 1**).

Considering that strip gradient and time strongly affected the overall structure of both bacterial and fungal communities, we split the dataset for individual sampling time and investigated the variation contributed by sampling locations, separately in leaf and soil. The visualization of distance matrices using PCoA analysis showed marked clustering of FB leaf microbial communities at the different sampling gradients at the individual sampling times, except for fungal communities at sampling time 3 (**Figure 4A,B**). PERMANOVA confirmed that

strip gradient had a pronounced effect on the leaf bacterial communities in FB at the three sampling time points (**Supplementary Table S10**). Also, sampling location had significant effect on fungal community at different sampling times. Generally, pairwise differences on community dissimilarity were more significant between sampling locations further apart and least to no effects on sampling gradients with least distances. Soil microbial beta diversity was less distinct with generally minor clustering between sampling locations at the different sampling times in both FB and SB (**Figure S14A,B, Supplementary Table S10**).

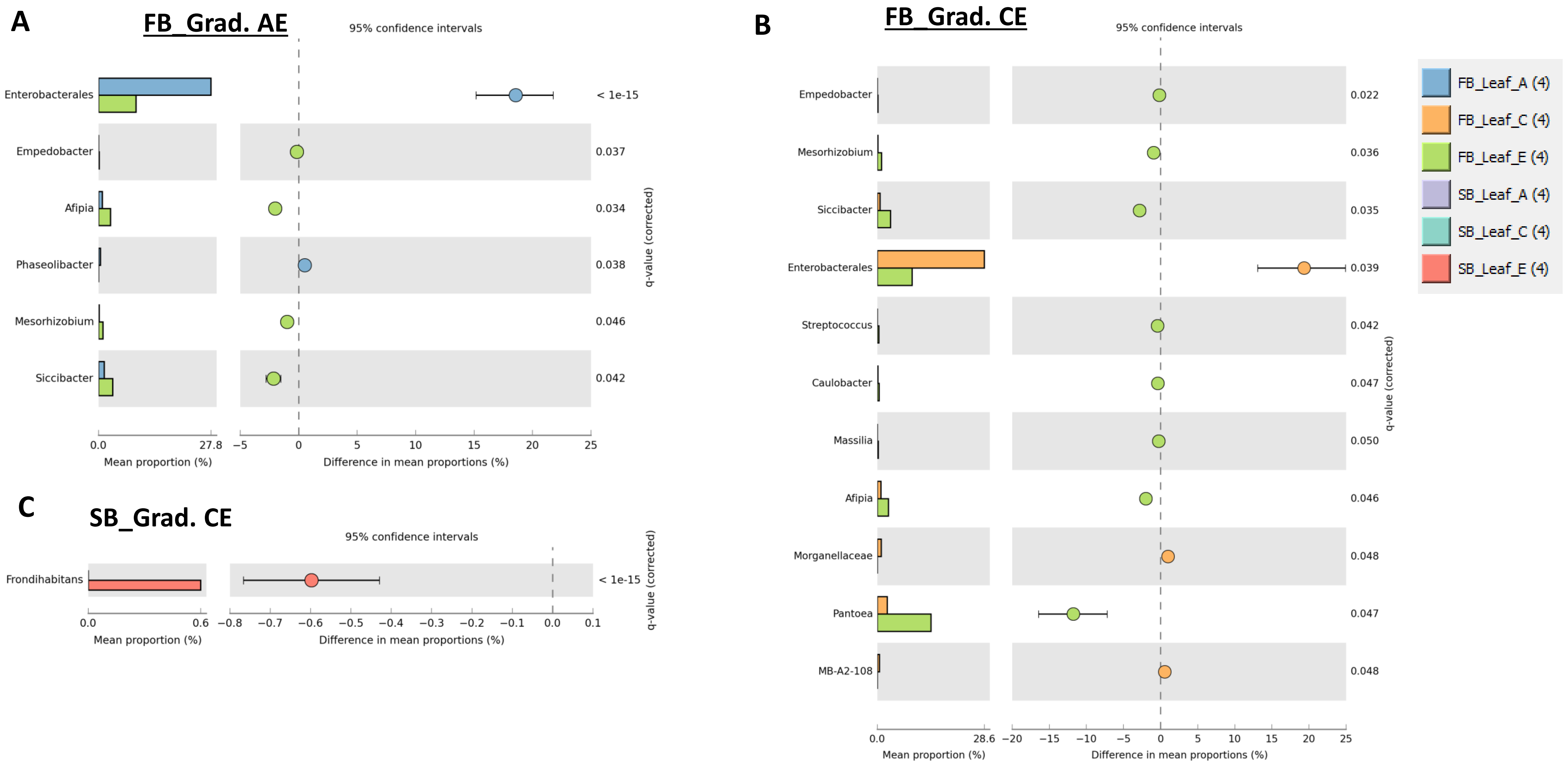


**Fig. S1:** Leaf bacterial genera with significantly different relative abundances between sampling locations A) A and E and B) C and E in FB, and C) C and E in SB at T2 in 2022. Analysis was performed with the STAMP software with White's nonparametric t test, followed by Benjamini-Hochberg correction (FDR) with q-value < 0.05. The statistical test used was two-sided.

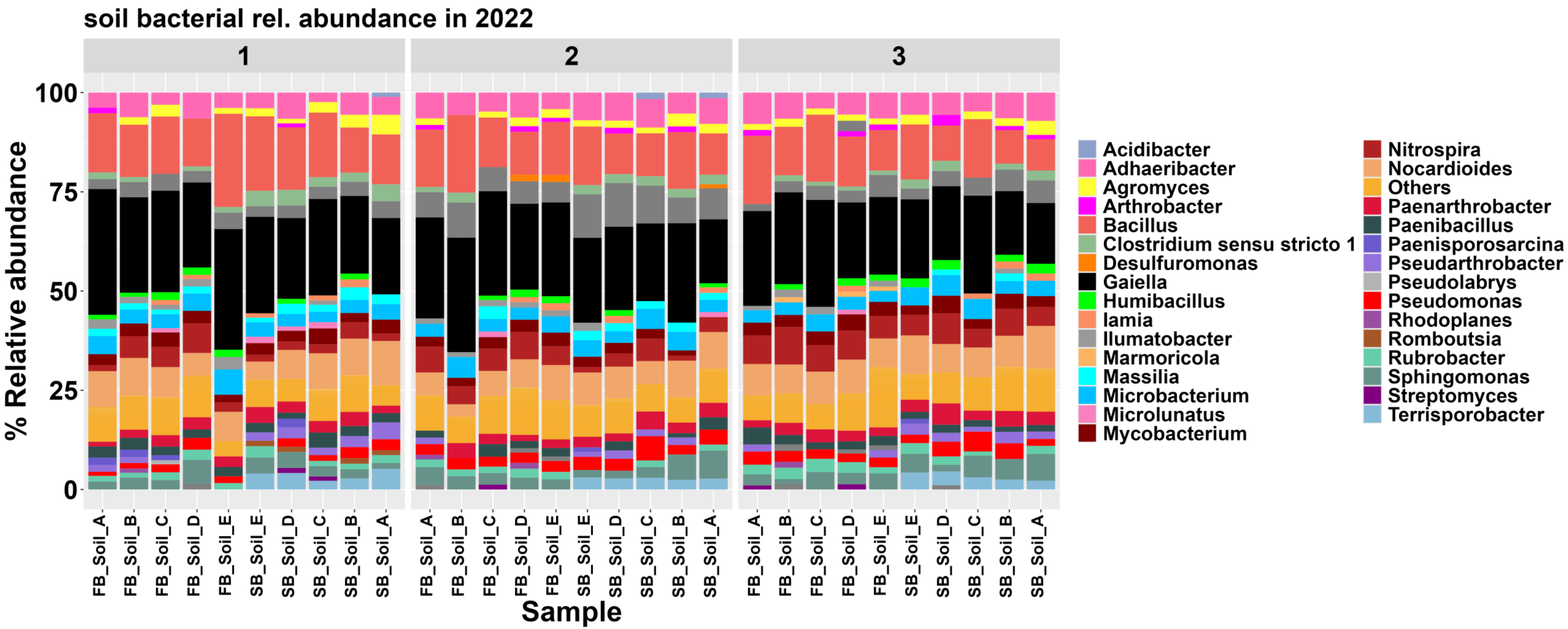


**Fig. S2:** Soil bacterial relative abundance in faba bean (FB) and spring barley (SB) samples across strip gradients (from centre of strip towards the edge border rows (A-E)) at different sampling times in 2022.

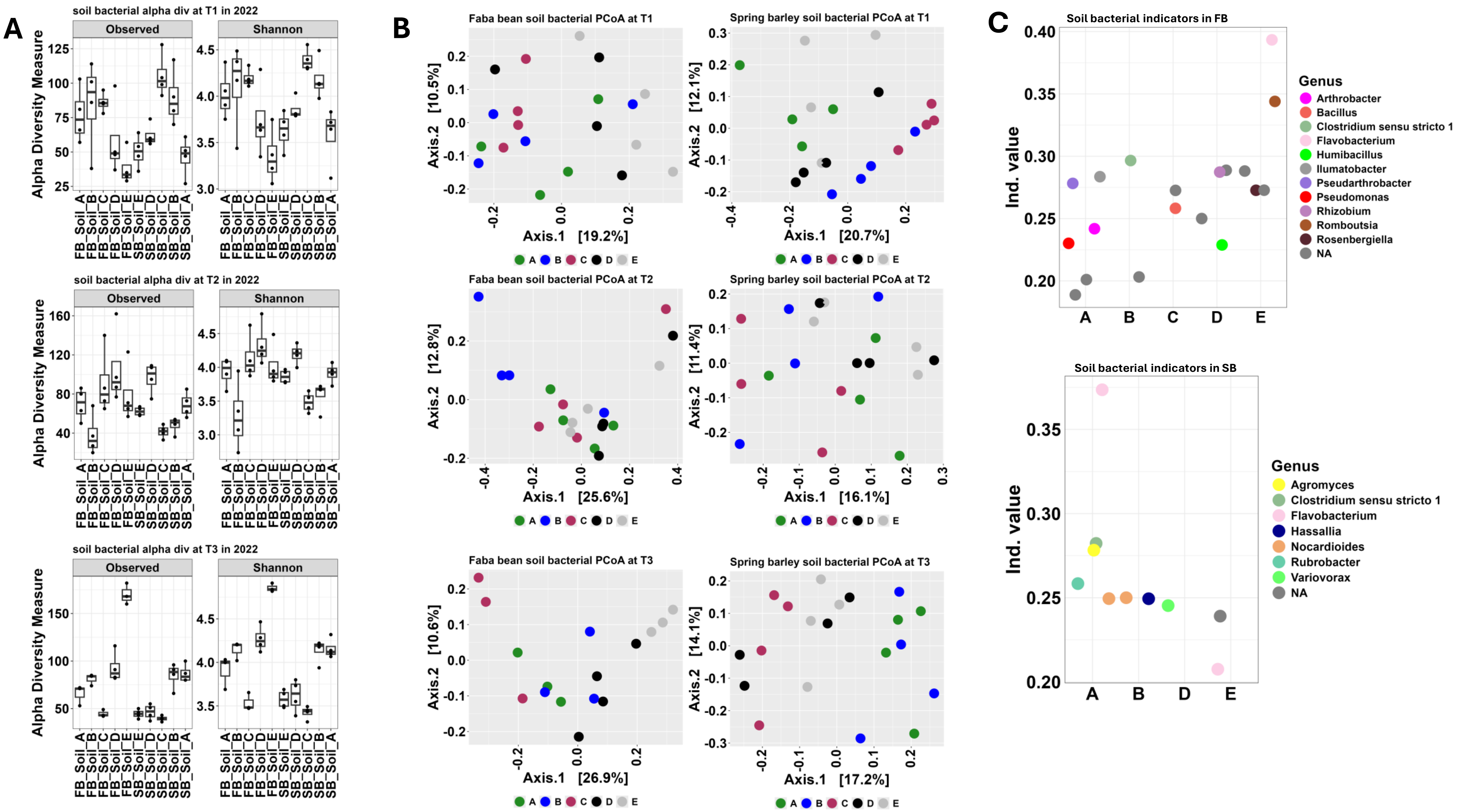


**Fig. S3:** A) Soil bacterial alpha diversity in faba bean (FB) and spring barley (SB) samples across strip gradients (from centre of strip towards the edge border rows (A-E)) at different sampling times in 2022. B) Principal coordinate analysis (PCoA) plots of soil bacterial communities at different sampling sites in FB and SB strips at different time points in 2022. C) Soil indicator bacterial species characterizing different gradient positions in FB and SB spring fields in 2022.

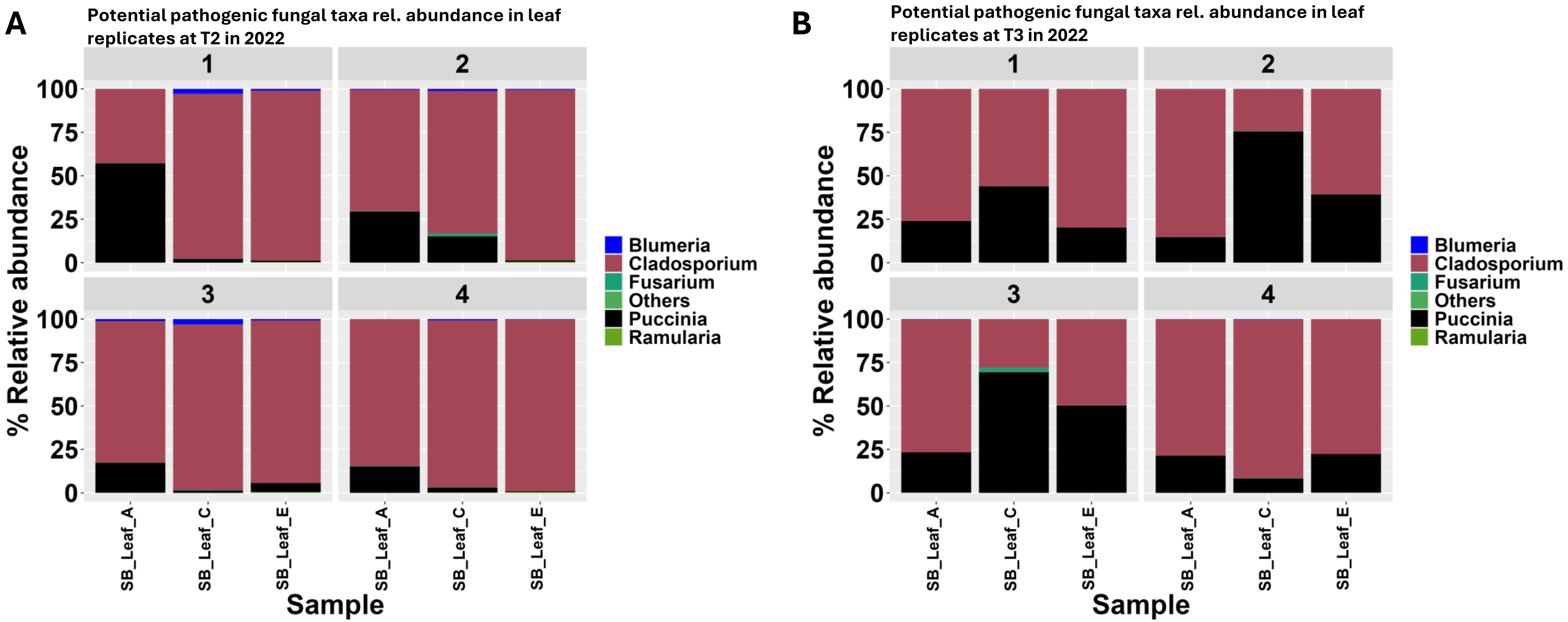


**Fig. S4:** Relative abundance of potential SB leaf fungal pathogenic genera in replicates at sampling times A) T2 and B) T3 in 2022.

**Fig. S5:** Relative abundance of soil fungi at sampling time A) 1, 2 and B) sampling time 3 in 2022.

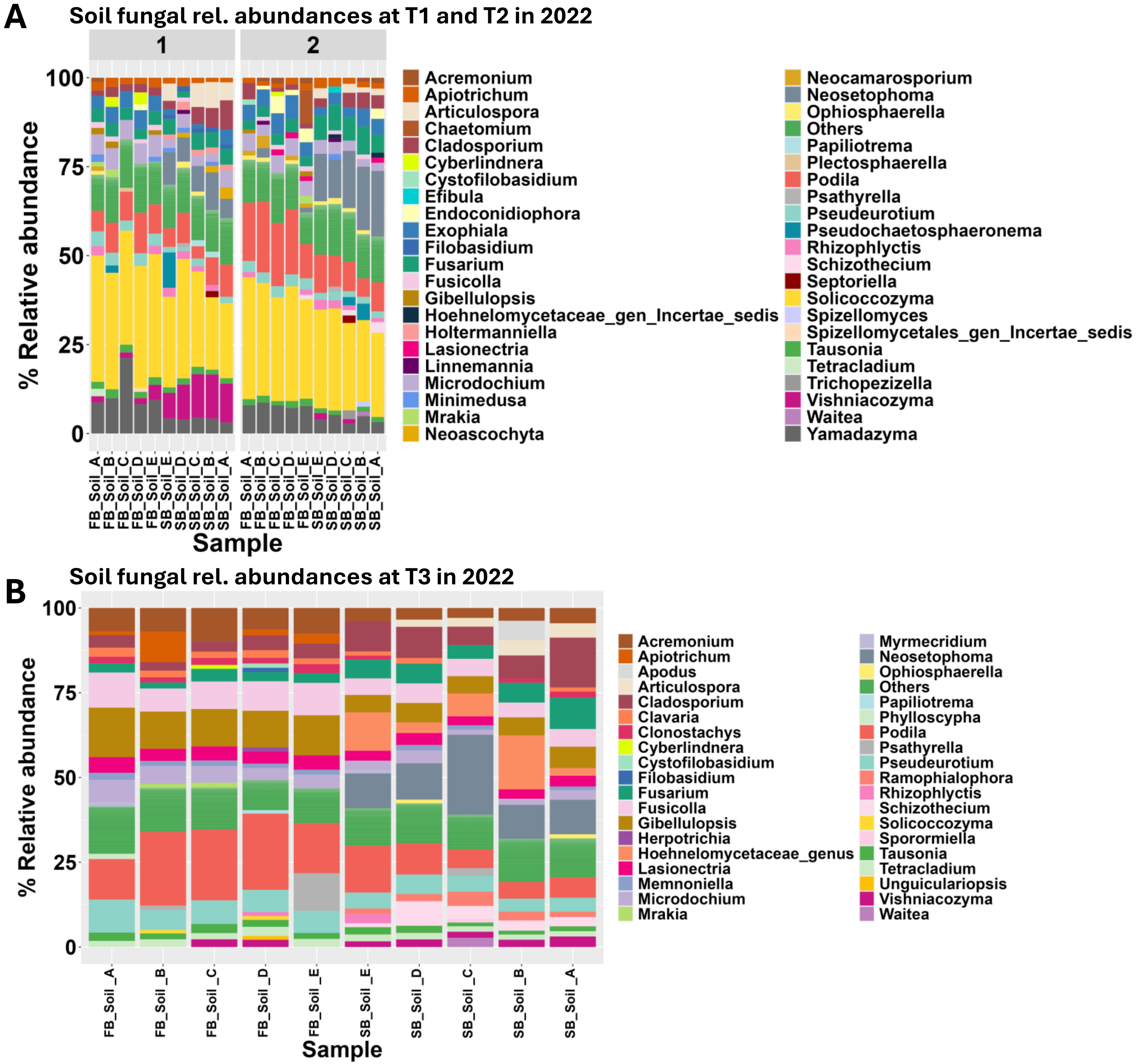

# TIME SPECIFIC

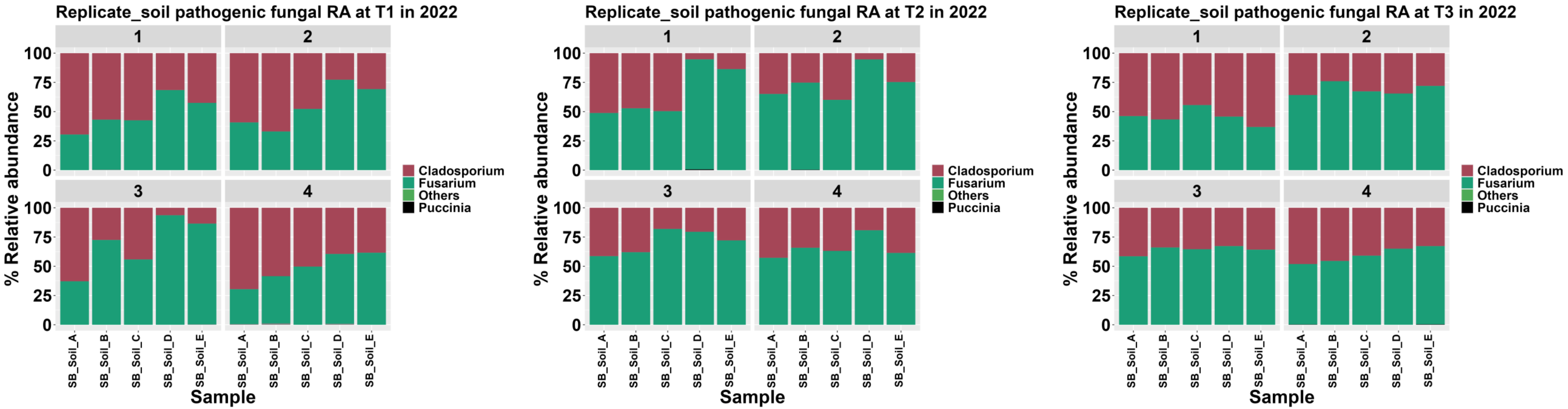


**Fig. S6:** Relative abundance of potential soil fungal pathogens in SB sample replicates for the different sampling time points in 2022.

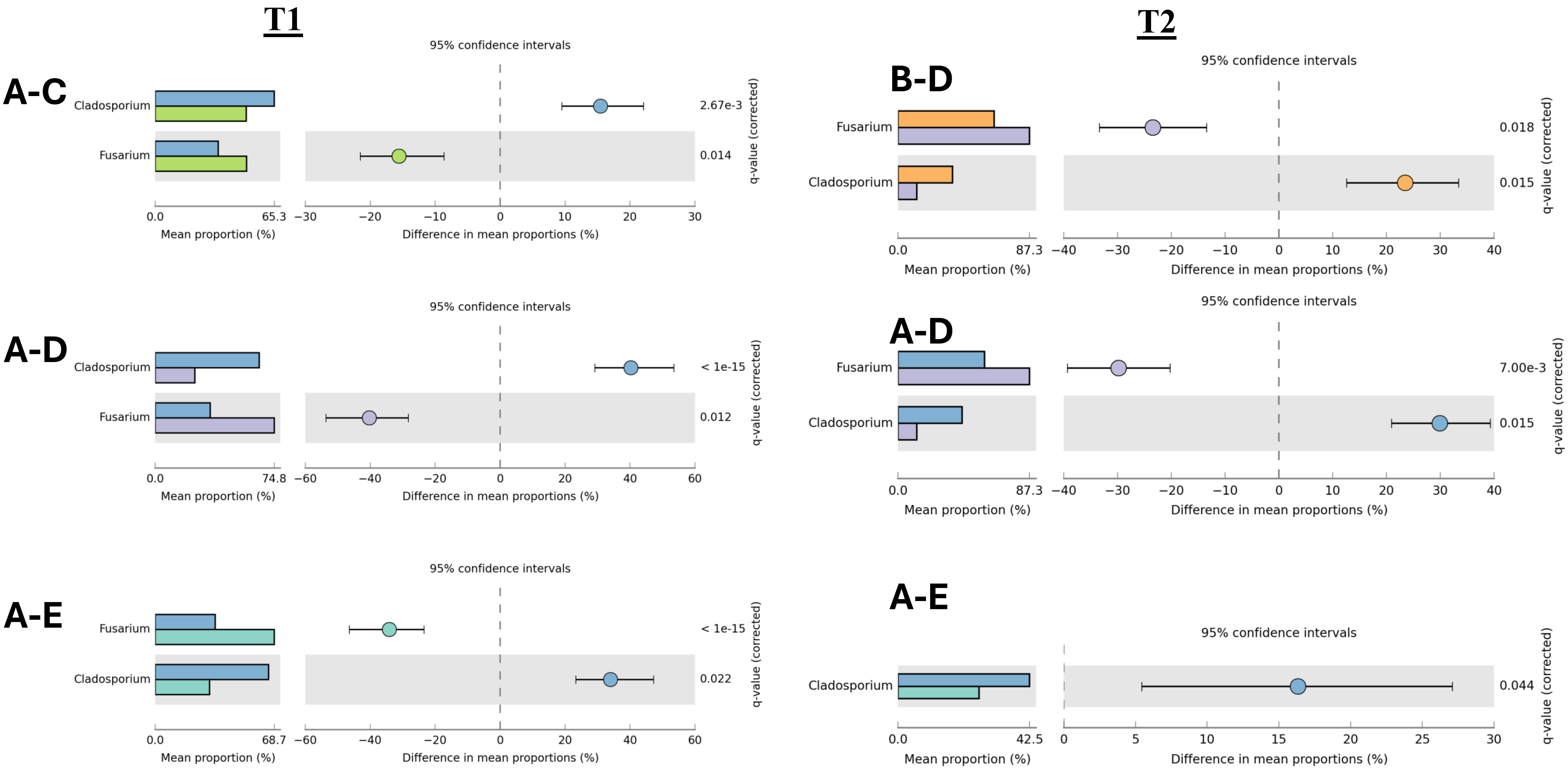


**Fig. S7**: Differences in the abundances of potential fungal pathogenic genera in soil between gradient positions at T1 and T2 in SB. Analysis was performed with the STAMP software with White's nonparametric t test, followed by Benjamini-Hochberg correction (FDR) with q-value < 0.05. The statistical test used was two-sided.

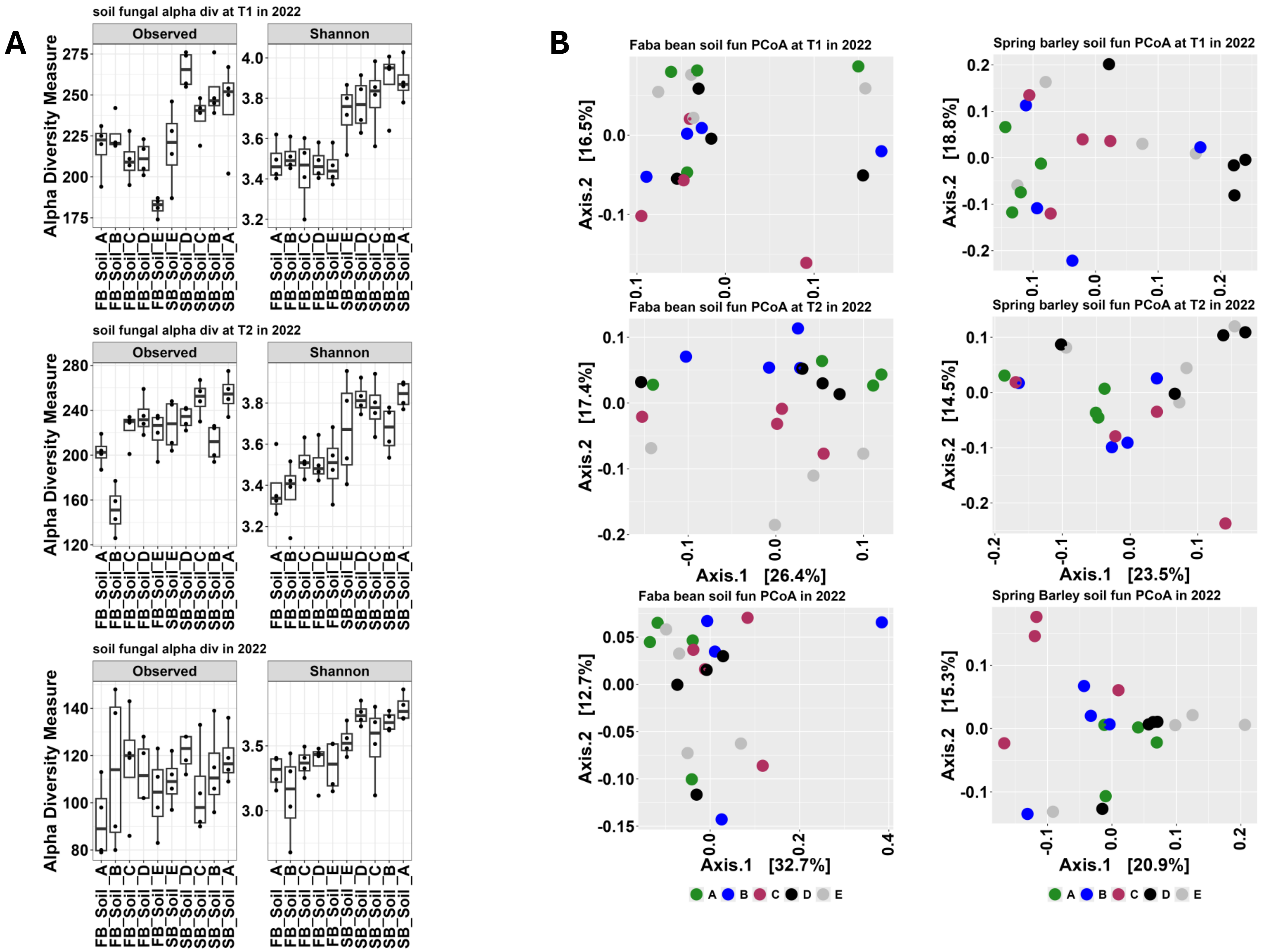


**Fig. S8**: A) Soil fungal alpha diversity in faba bean (FB) and spring barley (SB) samples across strip gradients (from centre of strip towards the edge border rows (A-E)) at different sampling times in 2022. B) Principal coordinate analysis (PCoA) plots of soil fungal communities at different sampling sites in FB and SB strips at different time points in 2022

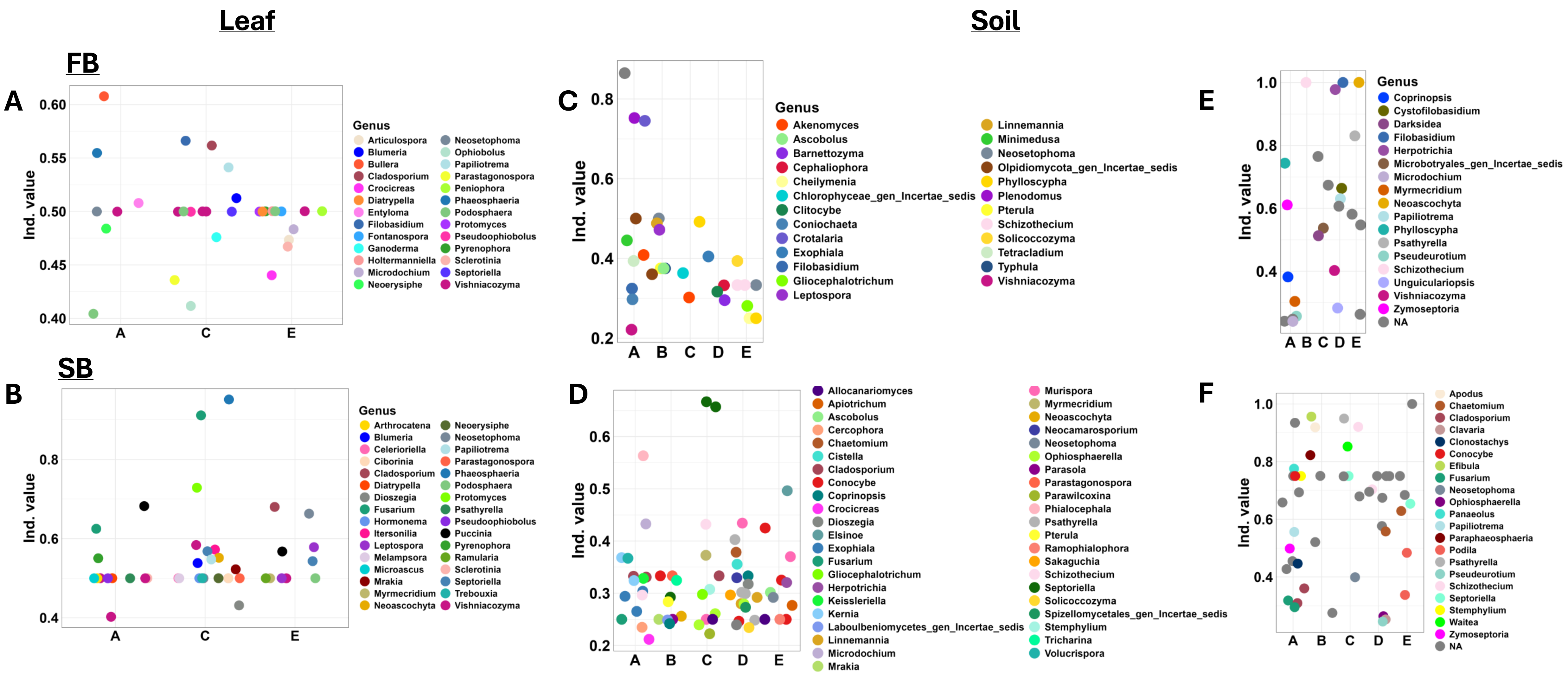


**Fig. S9:** Indicator leaf (A,B) and soil (C-F) fungal species characterizing different sampling locations in FB and SB spring fields in 2022. CD using T1, T2 and EF using T3 datasets, respectively.

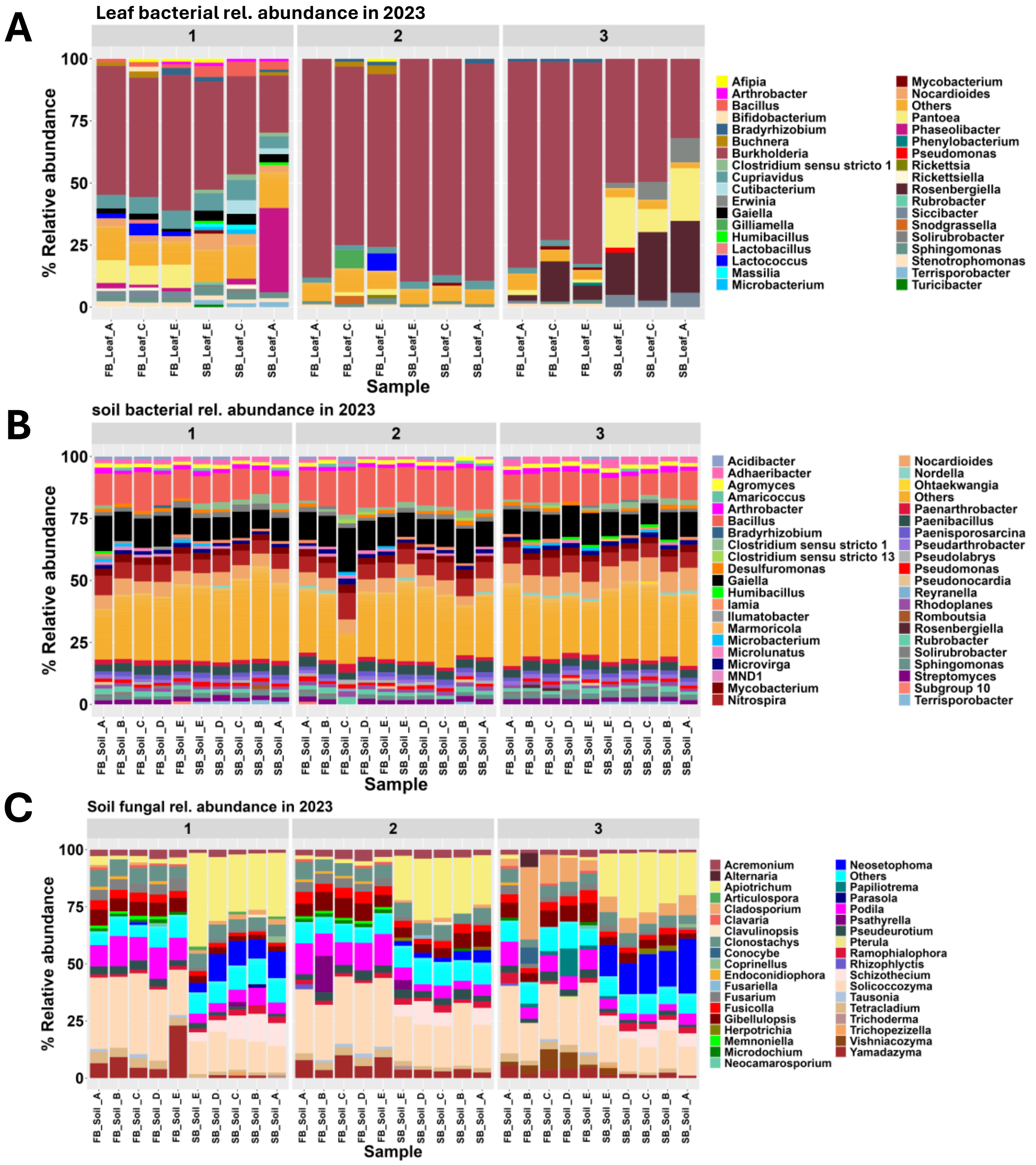


**Fig. S10:** A) Leaf bacterial, B) soil bacterial and C) soil fungal relative abundances in faba bean (FB) and spring barley (SB) samples across strip gradients (from centre of strip towards the edge border rows (A-E)) at different sampling times in 2023.

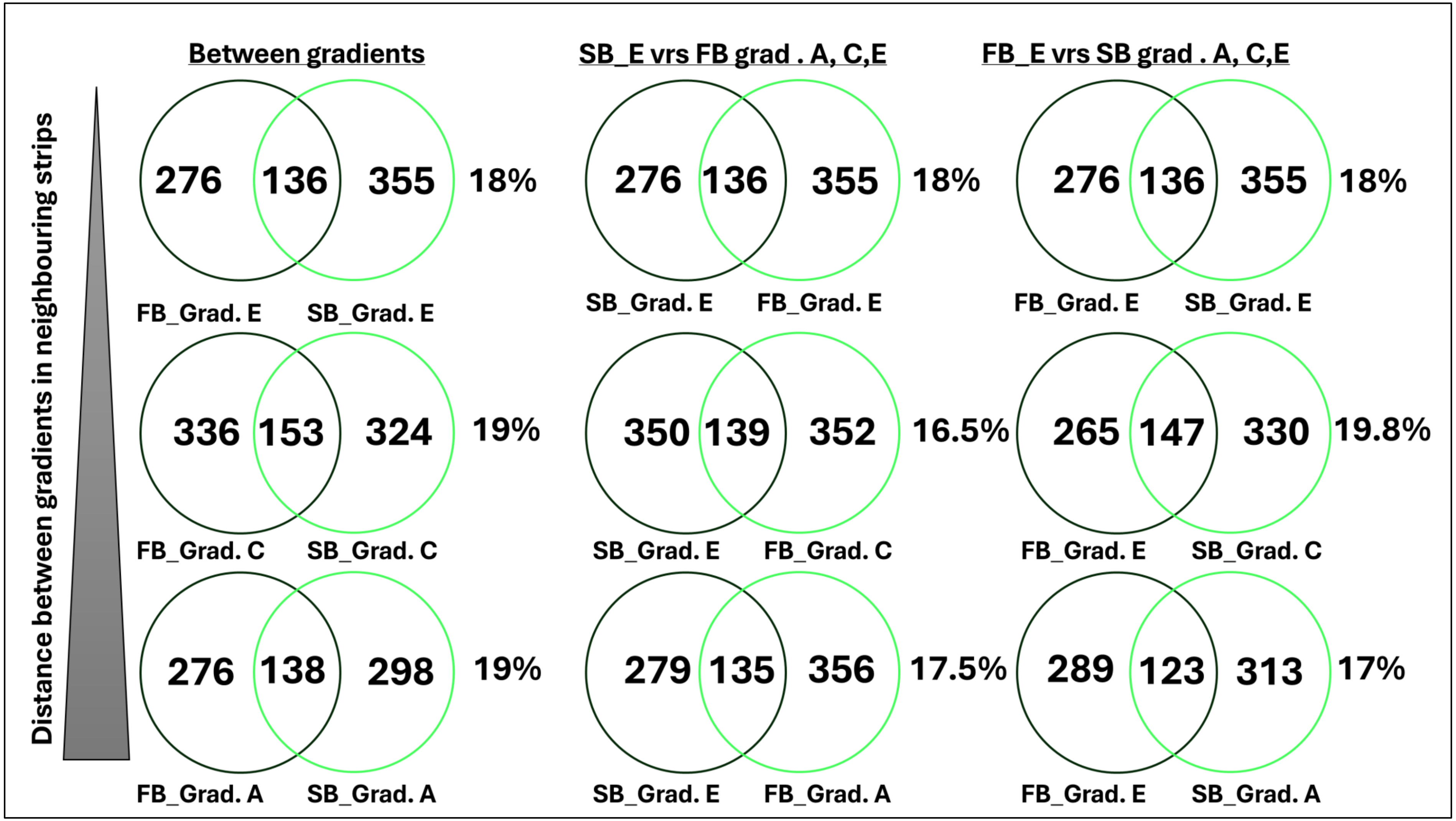


**Fig. S11:** Number of shared leaf bacterial taxa at different sampling locations within FB and SB in 2023.

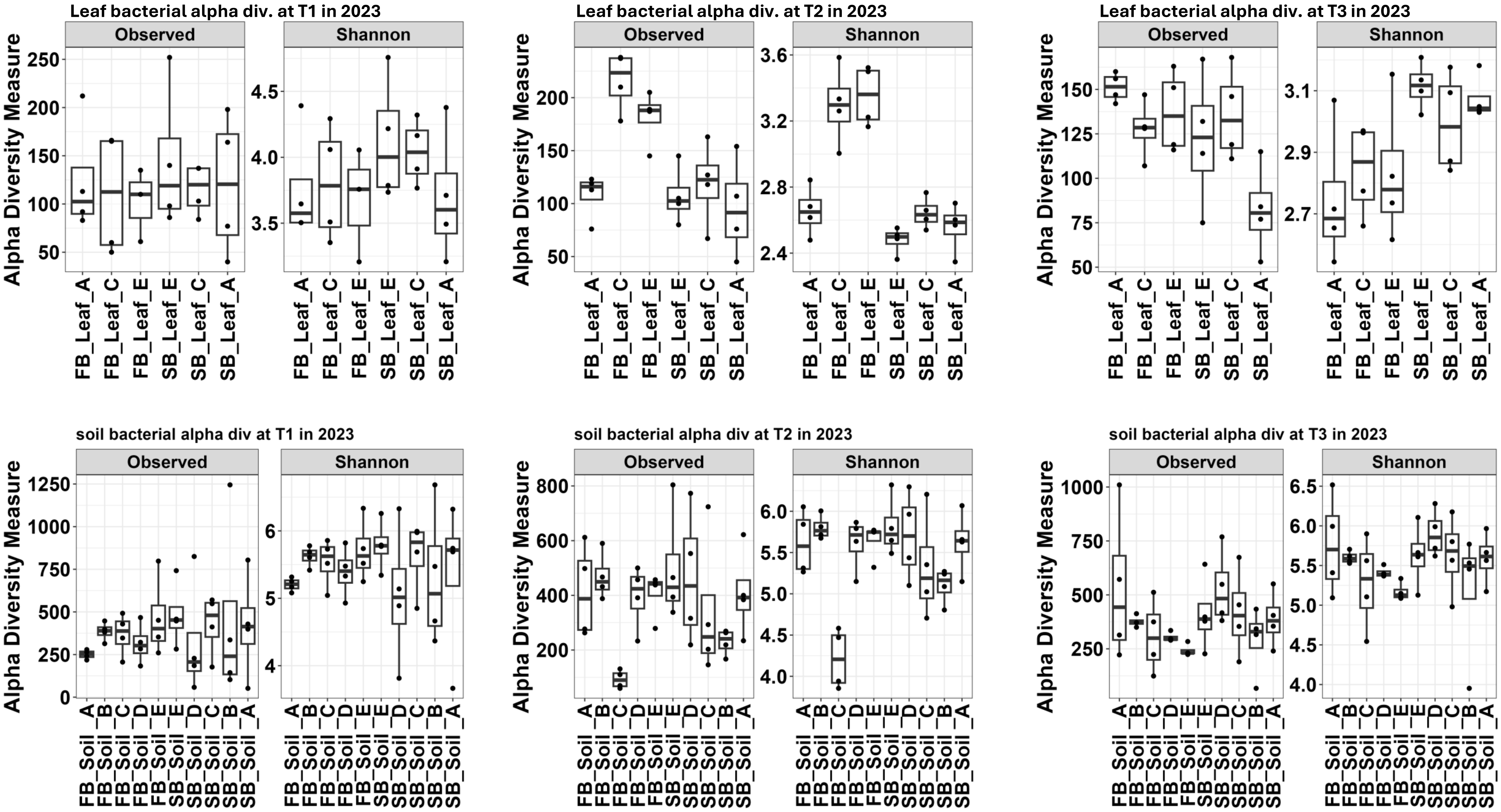


**Fig. S12:** A) Leaf and soil bacterial alpha diversity in faba bean (FB) and spring barley (SB) samples across strip gradients (from centre of strip towards the edge border rows (A-E)) at different sampling times in 2023.

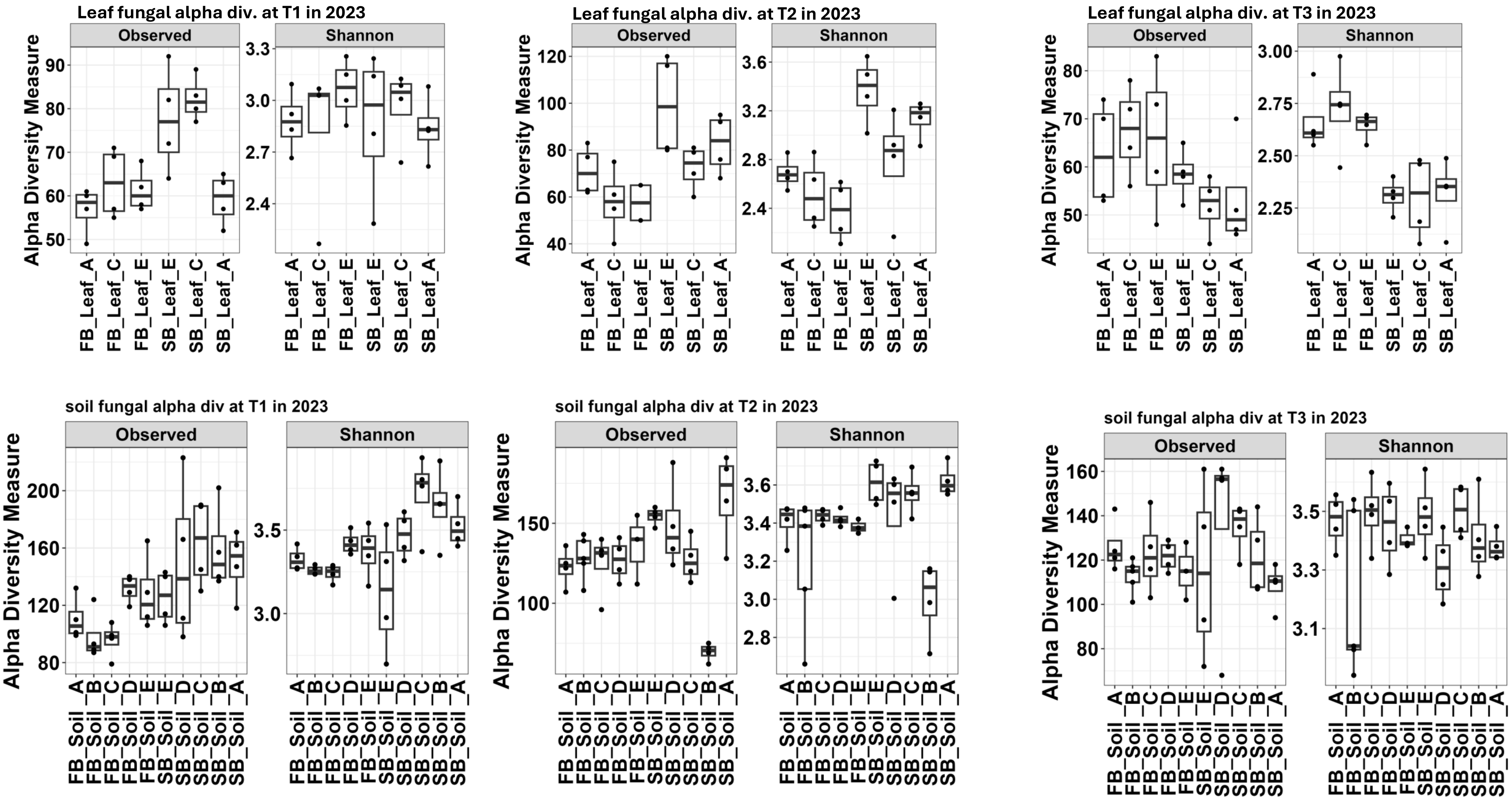


**Fig. S13:** A) Leaf and soil fungal alpha diversity in faba bean (FB) and spring barley (SB) samples across strip gradients (from centre of strip towards the edge border rows (A-E)) at different sampling times in 2023.

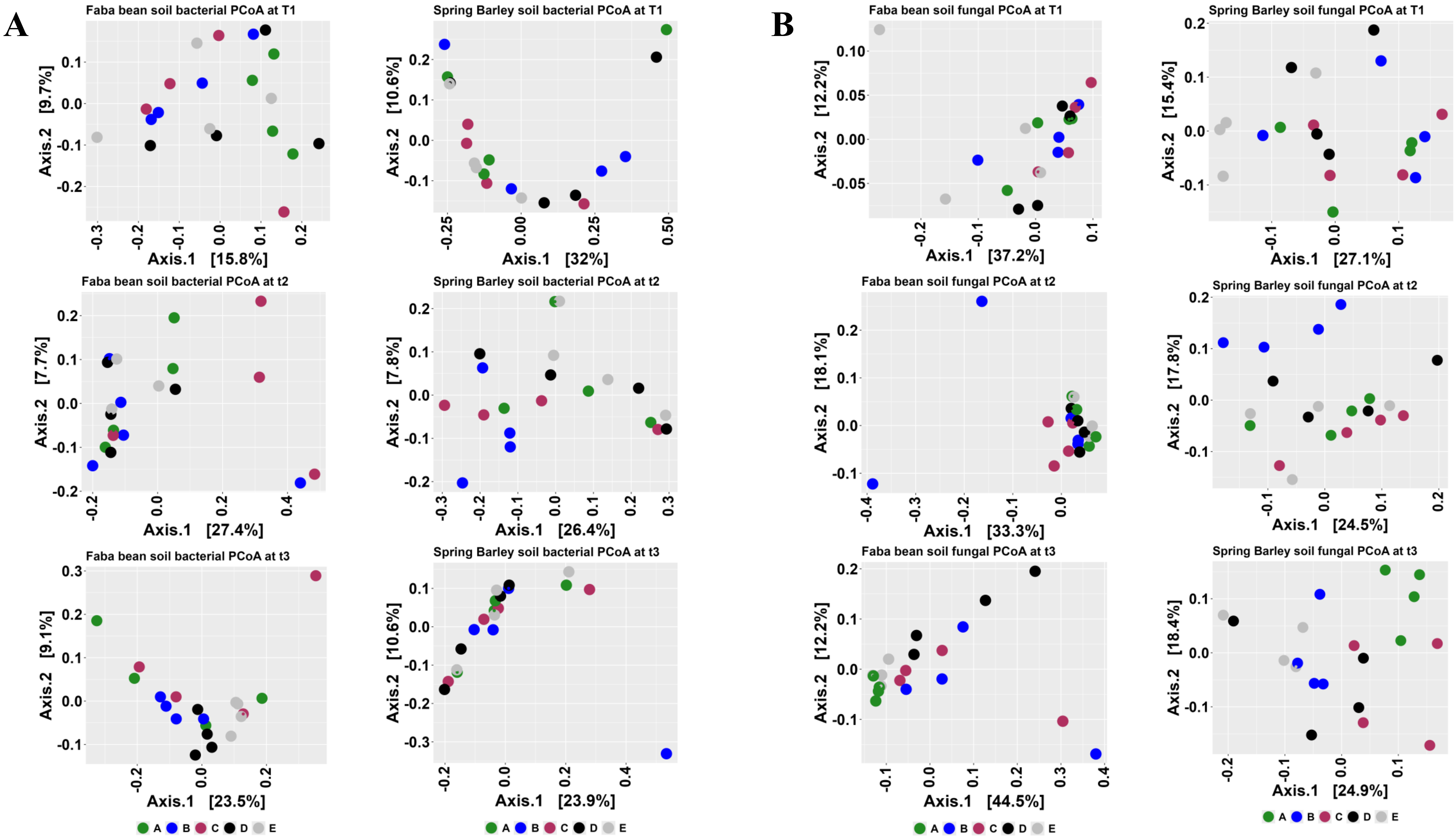


**Fig. S14:** Principal coordinate analysis (PCoA) plots of soil A) bacterial and B) fungal communities at different sampling sites in FB and SB strips at different time points in 2023.

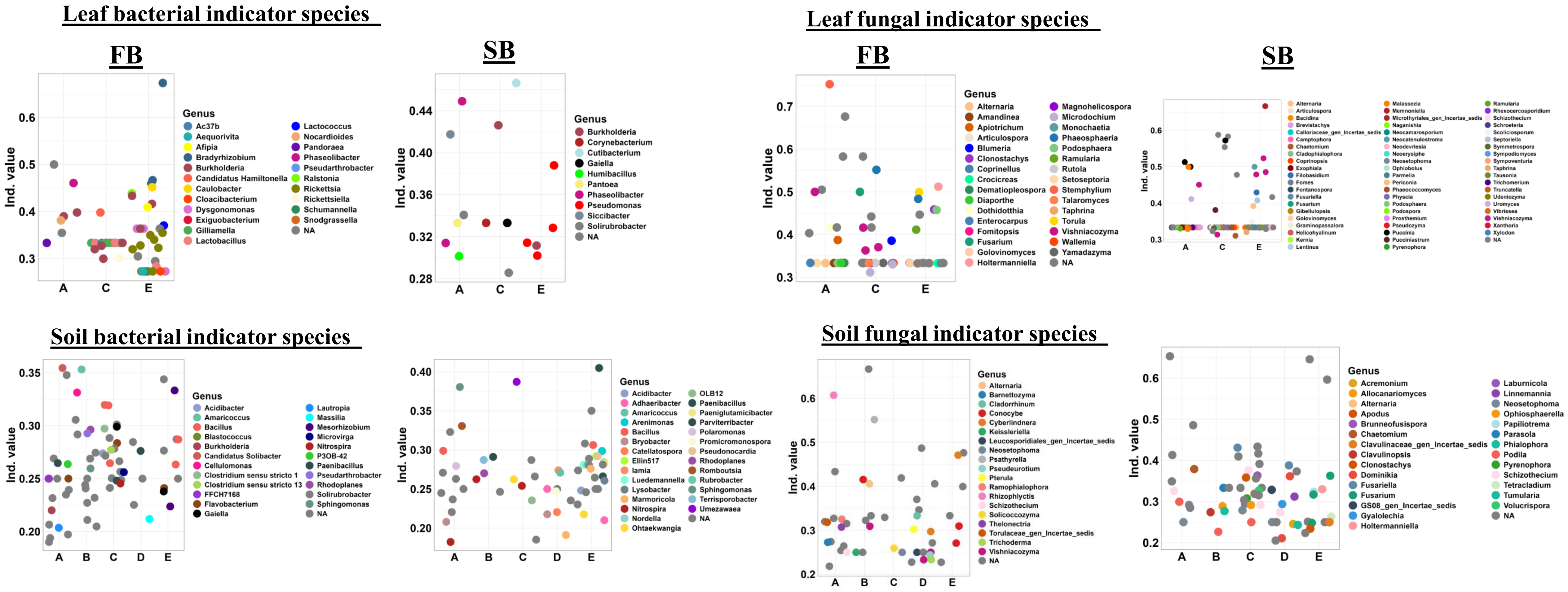


**Fig. S15:** Indicator bacterial and fungal species characterizing different leaf and soils at different sampling locations in FB and SB spring fields in 2023.

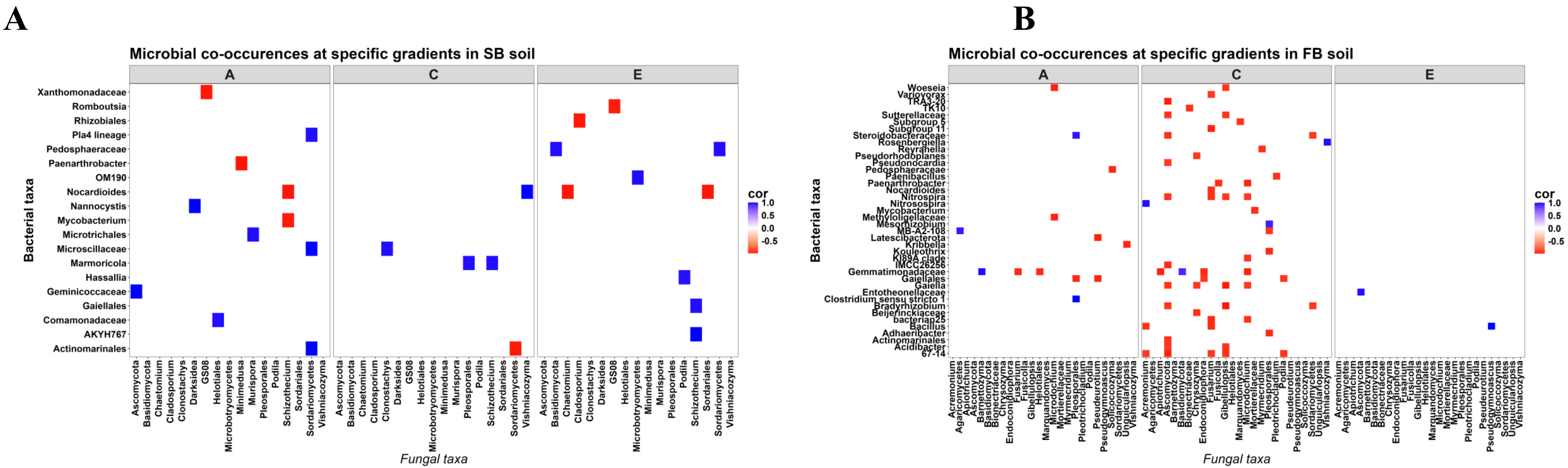


**Fig. S16:** Heat map showing significant correlations between bacterial and fungal taxa in A) SB and B) FB soil samples. Bacterial and fungal data from all the time points were pooled and used for the analysis. We used ASVs present in at least 10 samples with Spearman's rank correlations coefficients > 0.4 (positive correlations) or < −0.4 (negative correlations), with $p < 0.05$, and correction using Benjamini-Hochberg FDR. Positive and negative correlations are indicated by blue and red colours, respectively.

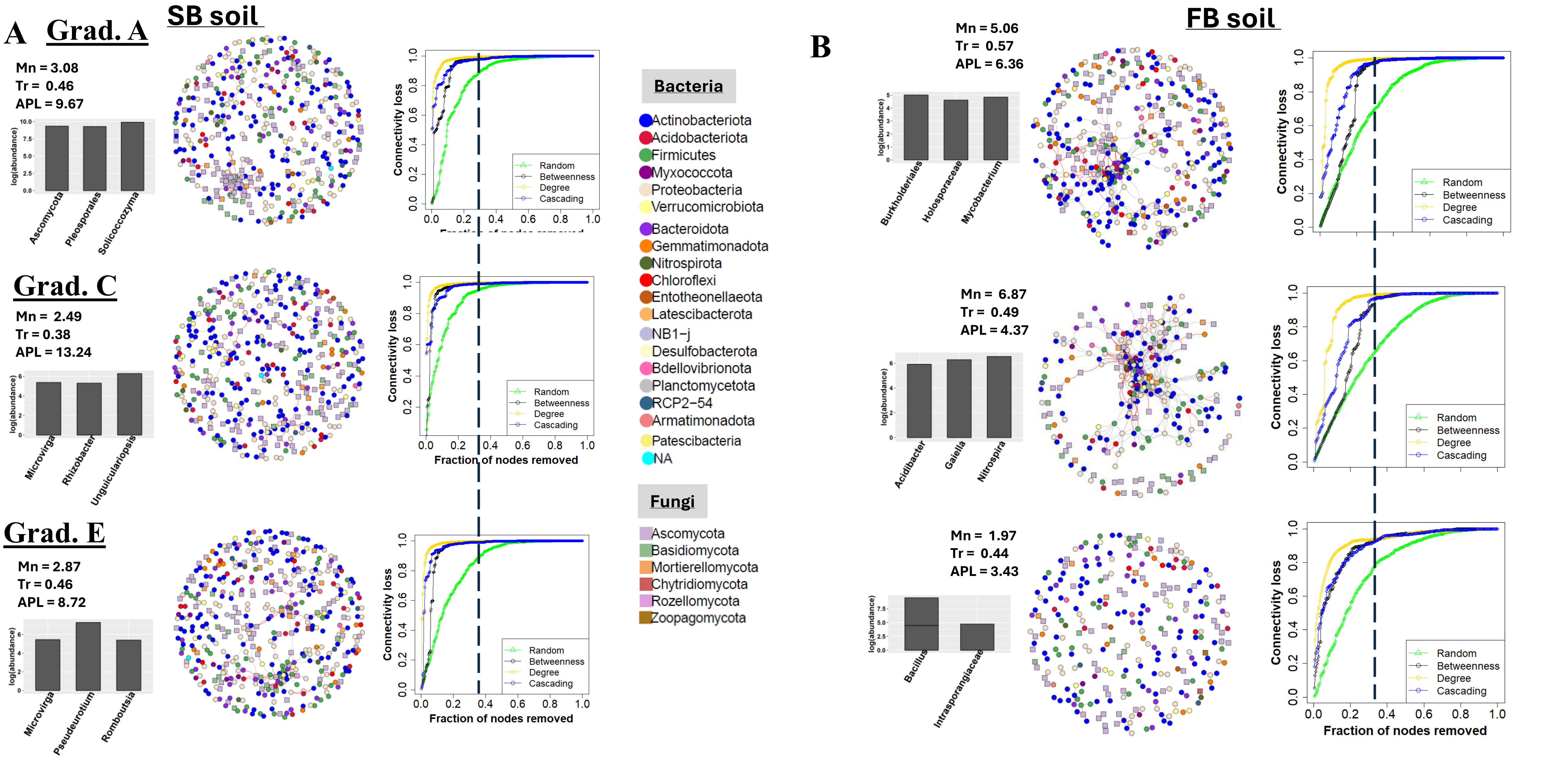


**Fig. S17:** Soil microbial co-occurrence networks computed for the different sampling locations (centre to edge sites) in A) SB and B) FB samples. The grey and red edges represent positive and negative correlations, respectively. Network tolerance to attack of the respective site-specific networks using change in connectivity as a function of the fraction of removed nodes. Dashed lines show the maximum fraction of nodes to be removed for 100% connectivity loss (highest break- down of network). The four sustained attack strategies to test tolerance included random removal, direct removal of nodes with highest betweenness centrality, target on nodes with the highest impact closeness (degree), and a combination of random and targeted on betweenness (cascading). Additionally, relative abundances of hub taxa identified (including the numbers of ASVs assigned to phyla) for each site-specific networks are shown.

**Table S2**: Sequence statistics including mean, median and range of reads per compartment for fungal and bacterial libraries at different days post inoculation (DPI) in 2022.

| | Sampling Time (T) | Compartment | Mean | Median | Min | Max | Total |
|---|---|---|---|---|---|---|---|
| Bacteria | 3 | Leaf | 9487 | 6554.5 | 1810 | 31841 | 227687 |
| | 1,2 and 3 | Soil | 4529.7 | 4223.5 | 1436 | 12018 | 525447 |
| Fungi | 2,3 | Leaf | 39050.5 | 37026.5 | 13769 | 80397 | 2499234 |
| | 1,2 | Soil | 40891.2 | 41289 | 15548 | 58417 | 3271294 |
| | 3 | Soil | 13702.7 | 12681.5 | 5991 | 28953 | 548108 |

**Table S3:** Sequence statistics including mean, median and range of reads per compartment for fungal and bacterial libraries at different days post inoculation (DPI) in 2023.

| | Sampling Time (T) | Compartment | Mean | Median | Min | Max | Total |
|---|---|---|---|---|---|---|---|
| Bacteria | 1,2,3 | Leaf | 10546.78 | 9178 | 1218 | 28743 | 748822 |
| | | Soil | 11560.27 | 10926.5 | 1066 | 43404 | 1387233 |
| Fungi | 1,2,3 | Leaf | 17097.88 | 14769 | 3681 | 49205 | 1231048 |
| | | Soil | 18401.76 | 17605.5 | 4754 | 48351 | 2208211 |

**Table S4:** Leaf bacterial alpha diversity pair wise comparison (using Wilcoxon rank sum test) between strip gradients (from middle of strip towards the edge border rows (A-E)) of faba bean (FB) and spring barley (SB) samples at different sampling time T3 in 2022.

| Compartment | Sampling gradient | Bacteria | |
|---|---|---|---|
| Leaf | | Observed | Shannon |
| SB p-value = 0.03877 | AXC | ns | ns |
| | AXE | 0.03877* | ns |
| | CXE | ns | ns |
| FB | AXC | ns | ns |
| | AXE | ns | ns |
| | CXE | ns | ns |

**Table S5:** Summary of permutational analysis of variance (PERMANOVA) and Pairwise Adonis at each sampling time in 2022 using the "adonis" test on Bray-Curtis distance matrices using bacterial and fungal community dissimilarity assessment using 1,000 permutations.

| Dataset | Time | Compartment | Factors | Bacteria (16S) $R^2$ | Fungi (ITS) $R^2$ |
|---|---|---|---|---|---|
| **Whole** | | | | | |
| FB | 1 | Leaf | Sampling location | na | na |
| | | Soil | Sampling location | 0.29** | ns |
| SB | 1 | Leaf | Sampling location | na | na |
| | | Soil | Sampling location | 0.32*** | 0.36** |
| FB | 2 | Leaf | Sampling location | na | 0.47*** |
| | | Soil | Sampling location | ns | 0.31* |
| SB | 2 | Leaf | Sampling location | na | 0.42*** |
| | | Soil | Sampling location | 0.26* | 0.32** |
| FB | 3 | Leaf | Sampling location | 0.48** | 0.43*** |
| | | Soil | Sampling location | ns | ns |
| SB | 3 | Leaf | Sampling location | 0.49** | 0.33* |
| | | Soil | Sampling location | ns | 0.40*** |
| **Pairwise** | | | | | |
| Faba bean | 1 | Leaf | A x C | na | na |
| | | | A x E | na | na |
| | | | C x E | na | na |
| | | Soil | | | |
| | | | A x B | ns | ns |
| | | | A x C | 0.22* | ns |
| | | | A x D | ns | ns |
| | | | A x E | 0.22* | ns |
| | | | B x C | ns | ns |
| | | | B x D | ns | ns |
| | | | B x E | ns | ns |
| | | | C x D | 0.23* | ns |
| | | | C x E | 0.25* | ns |
| | | | D x E | ns | ns |

| | | | | | |
|---|---|---|---|---|---|
| Spring barley (SB) | | Leaf | | | |
| | | | A x C | na | na |
| | | | A x E | na | na |
| | | | C x E | na | na |
| | | Soil | | | |
| | | | A x B | 0.24* | ns |
| | | | A x C | 0.35* | 0.25* |
| | | | A x D | ns | 0.43* |
| | | | A x E | ns | 0.25* |
| | | | B x C | ns | ns |
| | | | B x D | ns | ns |
| | | | B x E | 0.22* | ns |
| | | | C x D | 0.31* | 0.35* |
| | | | C x E | 0.29* | ns |
| | | | D x E | ns | ns |
| Faba bean (FB) | 2 | Leaf | | | |
| | | | A x C | na | 0.35* |
| | | | A x E | na | 0.55* |
| | | | C x E | na | ns |
| | | Soil | | | |
| | | | A x B | ns | ns |
| | | | A x C | ns | ns |
| | | | A x D | ns | ns |
| | | | A x E | ns | ns |
| | | | B x C | ns | 0.26* |
| | | | B x D | ns | ns |
| | | | B x E | ns | 0.32* |
| | | | C x D | ns | ns |
| | | | C x E | ns | ns |
| | | | D x E | ns | ns |
| Spring barley (SB) | | Leaf | | | |
| | | | A x C | na | 0.31* |
| | | | A x E | na | 0.35* |
| | | | C x E | na | 0.39* |
| | | Soil | | | |
| | | | A x B | ns | ns |
| | | | A x C | ns | ns |
| | | | A x D | ns | 0.31* |
| | | | A x E | ns | 0.27* |
| | | | Bx C | ns | ns |
| | | | B x D | ns | ns |
| | | | B x E | ns | ns |
| | | | Cx D | 0.25* | ns |

| | | | | | |
|---|---|---|---|---|---|
| | | | C x E | 0.21* | ns |
| | | | D xE | ns | ns |
| Faba bean (FB) | 3 | Leaf | | | |
| | | | A x C | ns | 0.35* |
| | | | A x E | 0.46* | 0.35* |
| | | | C x E | 0.52* | 0.37* |
| | | Soil | | | |
| | | | A x B | ns | ns |
| | | | A x C | ns | ns |
| | | | A x D | ns | ns |
| | | | A x E | ns | ns |
| | | | B x C | ns | ns |
| | | | B x D | ns | ns |
| | | | B x E | ns | ns |
| | | | C x D | ns | ns |
| | | | C x E | ns | ns |
| | | | D x E | ns | ns |
| Spring barley (SB) | | Leaf | | | |
| | | | A x C | 0.46* | ns |
| | | | A x E | 0.38* | 0.36* |
| | | | C x E | 0.42* | ns |
| | | Soil | | | |
| | | | A x B | ns | 0.30* |
| | | | A x C | ns | 0.35* |
| | | | A x D | ns | 0.33* |
| | | | A x E | ns | 0.29* |
| | | | B x C | ns | ns |
| | | | B x D | ns | 0.31* |
| | | | B x E | ns | ns |
| | | | C x D | ns | 0.31* |
| | | | C x E | ns | ns |
| | | | D x E | ns | ns |

Significance of test indicated as ***, p <0.001; **, p <0.01; *, p <0.05. The ns denotes not statistically significant and $R^2$ is the proportion of variation explained. The na denotes not analyzed.

**Table S6**: Soil bacterial and fungal alpha diversity pair wise comparison (using Wilcoxon rank sum test) between strip gradients (from middle of strip towards the edge border rows (A-E)) of faba bean (FB) and spring barley (SB) samples at different sampling time T2 and T3 in 2022.

| Compartment | Sampling location | Bacteria | | Fungi | |
|---|---|---|---|---|---|
| Sampling time 1 | | | | | |

| Soil | | Observed p-value = 0.005037 | Shannon p-value = 0.005102 | Observed | Shannon |
|---|---|---|---|---|---|
| SB | AXB | ns | ns | ns | ns |
| | AXC | 0.02077864 | 0.02304980 | ns | ns |
| | AXD | ns | ns | ns | ns |
| | AXE | ns | ns | ns | ns |
| | BXC | ns | ns | ns | ns |
| | BXD | ns | ns | ns | ns |
| | BXE | ns | ns | ns | ns |
| | CXD | ns | ns | ns | ns |
| | CXE | 0.01868675 | 0.01704050 | | ns |
| | DXE | ns | ns | 0.03061119 | ns |
| | | p-value = 0.05849 | p-value = 0.04745 | | |
| FB | AXB | ns | ns | ns | ns |
| | AXC | ns | ns | ns | ns |
| | AXD | ns | ns | ns | ns |
| | AXE | ns | ns | 0.03902581 | ns |
| | BXC | ns | ns | ns | ns |
| | BXD | ns | ns | ns | ns |
| | BXE | ns | ns | 0.02535373 | ns |
| | CXD | ns | ns | ns | ns |
| | CXE | 0.08372566 | 0.06036899 | ns | ns |
| | DXE | ns | ns | ns | ns |
| Sampling time 2 | | | | | |
| Soil | | p-value = 0.002399 | p-value = 0.002543 | | |
| SB | AXB | ns | ns | 0.04109554 | ns |
| | AXC | ns | ns | | ns |
| | AXD | ns | ns | | ns |
| | AXE | ns | ns | | ns |
| | BXC | | | 0.04653044 | ns |
| | BXD | 0.012676867 | 0.011524901 | ns | ns |
| | BXE | | | ns | ns |
| | CXD | 0.003749106 | 0.004220074 | ns | ns |
| | CXE | ns | ns | ns | ns |
| | DXE | ns | ns | ns | ns |
| | | p-value = 0.04258 | p-value = 0.05339 | | |
| FB | AXB | ns | ns | ns | ns |
| | AXC | ns | ns | ns | ns |
| | AXD | ns | ns | ns | ns |
| | AXE | ns | ns | ns | ns |

| | | | | | |
|---|---|---|---|---|---|
| | BXC | ns | ns | 0.04639784 | ns |
| | BXD | 0.02786847 | 0.0340810 | 0.02069795 | ns |
| | BXE | ns | ns | 0.03676397 | ns |
| | CXD | ns | ns | ns | ns |
| | CXE | ns | ns | ns | ns |
| | DXE | ns | ns | ns | ns |
| Sampling time 3 | | | | | |
| soil | | p-value = 0.005202 | p-value = 0.004144 | | |
| SB | AXB | | | ns | ns |
| | AXC | 0.01513975 | 0.01152490 | ns | ns |
| | AXD | ns | ns | ns | ns |
| | AXE | ns | ns | ns | ns |
| | BXC | 0.03027950 | 0.01538320 | ns | ns |
| | BXD | ns | ns | ns | ns |
| | BXE | ns | ns | ns | ns |
| | CXD | ns | ns | ns | ns |
| | CXE | ns | ns | ns | ns |
| | DXE | ns | ns | ns | ns |
| | | p-value = 0.008676 | p-value = 0.009445 | | |
| FB | AXB | ns | ns | ns | ns |
| | AXC | ns | ns | ns | ns |
| | AXD | ns | ns | ns | ns |
| | AXE | ns | ns | ns | ns |
| | BXC | ns | ns | ns | ns |
| | BXD | ns | ns | ns | ns |
| | BXE | ns | ns | ns | ns |
| | CXD | ns | ns | ns | ns |
| | CXE | 0.008251356 | 0.008251356 | ns | ns |
| | DXE | ns | ns | ns | ns |

**Table S7**: Fungal leaf alpha diversity pair wise comparison (using Wilcoxon rank sum test) between strip gradients (from middle of strip towards the edge border rows (A-E)) of faba bean (FB) and spring barley (SB) samples at different sampling time T2 and T3 in 2022.

| Compartment | Sampling location | Fungi | |
|---|---|---|---|
| Sampling time 2 | | | |
| Leaf | | Observed | Shannon p-value = 0.04884 |

| | | | |
|---|---|---|---|
| SB | AXC | ns | ns |
| | AXE | ns | ns |
| | CXE | ns | ns |
| FB | AXC | ns | ns |
| | AXE | ns | 0.04268439 |
| | CXE | ns | ns |
| | | | |
| Sampling time 3 | | | |
| Leaf | | | |
| SB | AXC | ns | ns |
| | AXE | ns | ns |
| | CXE | ns | ns |
| FB p-value = 0.01729 | AXC | ns | ns |
| | AXE | ns | 0.01337858 |
| | CXE | ns | ns |

**Table S8:** Leaf alpha diversity pair wise comparison (using Wilcoxon rank sum test) between strip gradients (from middle of strip towards the edge border rows (A-E)) of faba bean (FB) and spring barley (SB) samples at different sampling times in 2023.

| Compartment | Sampling location | Bacteria | | Fungi | |
|---|---|---|---|---|---|
| Sampling time 1 | | | | | |
| Leaf | | Observed | Shannon | Observed p-value = 0.03455 | Shannon |
| SB | AXC | ns | ns | 0.04268439 | |
| | AXE | ns | ns | ns | ns |
| | CXE | ns | ns | ns | ns |
| FB | AXC | ns | ns | ns | ns |
| | AXE | ns | ns | ns | ns |
| | CXE | ns | ns | ns | ns |
| | | | | | |
| Sampling time 2 | | | | | |
| Leaf | | | | | |
| SB | AXC | | | ns | ns |
| | AXE | | | ns | ns |
| | CXE | | | ns | ns |
| | | p-value = 0.0154 | p-value = 0.02491 | ns | ns |
| FB | AXC | 0.01337858 | 0.02790439 | ns | ns |

| | | | | | |
|---|---|---|---|---|---|
| | AXE | | | ns | ns |
| | CXE | | | ns | ns |
| | | | | | |
| Sampling time 3 | | | | | |
| Leaf | | | | | |
| SB | AXC | ns | ns | ns | ns |
| | AXE | ns | ns | ns | ns |
| | CXE | ns | ns | ns | ns |
| FB | AXC | ns | ns | ns | ns |
| | AXE | ns | ns | ns | ns |
| | CXE | ns | ns | ns | ns |

**Table S9:** Soil alpha diversity pair wise comparison (using Wilcoxon rank sum test) between strip gradients (from middle of strip towards the edge border rows (A-E)) of faba bean (FB) and spring barley (SB) samples at different sampling times in 2023.

| Compartment | Sampling location | Bacteria | | Fungi | |
|---|---|---|---|---|---|
| Sampling time 1 | | | | | |
| Soil | | Observed | Shannon | Observed | Shannon |
| SB | AXB | ns | ns | ns | ns |
| | AXC | ns | ns | ns | ns |
| | AXD | ns | ns | ns | ns |
| | AXE | ns | ns | ns | ns |
| | BXC | ns | ns | ns | ns |
| | BXD | ns | ns | ns | ns |
| | BXE | ns | ns | ns | ns |
| | CXD | ns | ns | ns | ns |
| | CXE | ns | ns | ns | ns |
| | DXE | ns | ns | ns | ns |
| | | | | p-value = 0.02941 | |
| FB | AXB | ns | ns | ns | ns |
| | AXC | ns | ns | ns | ns |
| | AXD | ns | ns | ns | ns |
| | AXE | ns | ns | ns | ns |
| | BXC | ns | ns | ns | ns |
| | BXD | ns | ns | ns | ns |
| | BXE | ns | ns | ns | ns |
| | CXD | ns | ns | ns | ns |
| | CXE | ns | ns | ns | ns |
| | DXE | ns | ns | ns | ns |

| | | | | | |
|---|---|---|---|---|---|
| Sampling time 2 | | | | p-value = 0.009961 | |
| Soil | | | | | |
| SB | AXB | ns | ns | 0.01538320 | |
| | AXC | ns | ns | ns | ns |
| | AXD | ns | ns | ns | ns |
| | AXE | ns | ns | ns | ns |
| | BXC | ns | ns | ns | ns |
| | BXD | ns | ns | ns | ns |
| | BXE | ns | ns | 0.02486425 | ns |
| | CXD | ns | ns | ns | ns |
| | CXE | ns | ns | ns | ns |
| | DXE | ns | ns | ns | ns |
| | | | p-value = 0.04916 | | |
| FB | AXB | ns | ns | ns | ns |
| | AXC | ns | ns | ns | ns |
| | AXD | ns | ns | ns | ns |
| | AXE | ns | ns | ns | ns |
| | BXC | ns | ns | ns | ns |
| | BXD | ns | ns | ns | ns |
| | BXE | ns | ns | ns | ns |
| | CXD | ns | ns | ns | ns |
| | CXE | ns | ns | ns | ns |
| | DXE | ns | ns | ns | ns |
| Sampling time 3 | | | | | |
| Soil | | | | | |
| SB | AXB | ns | ns | ns | ns |
| | AXC | ns | ns | ns | ns |
| | AXD | ns | ns | ns | ns |
| | AXE | ns | ns | ns | ns |
| | BXC | ns | ns | ns | ns |
| | BXD | ns | ns | ns | ns |
| | BXE | ns | ns | ns | ns |
| | CXD | ns | ns | ns | ns |
| | CXE | ns | ns | ns | ns |
| | DXE | ns | ns | ns | ns |
| FB | AXB | ns | ns | ns | ns |
| | AXC | ns | ns | ns | ns |
| | AXD | ns | ns | ns | ns |
| | AXE | ns | ns | ns | ns |
| | BXC | ns | ns | ns | ns |
| | BXD | ns | ns | ns | ns |

| | | | | | |
|---|---|---|---|---|---|
| | BXE | ns | ns | ns | ns |
| | CXD | ns | ns | ns | ns |
| | CXE | ns | ns | ns | ns |
| | DXE | ns | ns | ns | ns |

**Table S10:** Summary of permutational analysis of variance (PERMANOVA) and Pairwise Adonis at each sampling time in 2023 using the "adonis" test on Bray-Curtis distance matrices using bacterial and fungal community dissimilarity assessment using 1,000 permutations.

| **Dataset** | **Time** | **Compartment** | **Factors** | **Bacteria (16S) $R^2$** | **Fungi (ITS) $R^2$** |
|---|---|---|---|---|---|
| **Whole** | | | | | |
| FB | 1 | Leaf | Sampling location | 0.40*** | 0.41*** |
| | | Soil | Sampling location | ns | 0.29* |
| SB | 1 | Leaf | Sampling location | 0.51*** | 0.37*** |
| | | Soil | Sampling location | ns | 0.35*** |
| FB | 2 | Leaf | Sampling location | 0.43*** | 0.36*** |
| | | Soil | Sampling location | 0.41* | 0.31*** |
| SB | 2 | Leaf | Sampling location | ns | 0.38*** |
| | | Soil | Sampling location | ns | 0.38*** |
| FB | 3 | Leaf | Sampling location | 0.45** | ns |
| | | Soil | Sampling location | ns | 0.36* |
| SB | 3 | Leaf | Sampling location | ns | 0.35* |
| | | Soil | Sampling location | ns | 0.42*** |
| **Pairwise** | | | | | |
| Faba bean (FB) | Time 1 | Leaf | | | |
| | | | A x C | 0.28* | 0.35* |
| | | | A x E | 0.40* | 0.32* |
| | | | C x E | 0.33* | 0.36* |
| | | Soil | | | |
| | | | A x B | ns | ns |
| | | | A x C | ns | ns |
| | | | A x D | ns | ns |
| | | | A x E | ns | ns |

| | | | | | |
|---|---|---|---|---|---|
| | | | B x C | ns | ns |
| | | | B x D | ns | ns |
| | | | B x E | ns | ns |
| | | | C x D | ns | ns |
| | | | C x E | ns | 0.32* |
| | | | D x E | ns | ns |
| Spring barley (SB) | | Leaf | | | |
| | | | A x C | 0.50* | 0.34* |
| | | | A x E | 0.48* | 0.29* |
| | | | C x E | 0.25* | 0.29* |
| | | Soil | | | |
| | | | A x B | ns | ns |
| | | | A x C | ns | ns |
| | | | A x D | ns | ns |
| | | | A x E | ns | ns |
| | | | B x C | ns | ns |
| | | | B x D | ns | ns |
| | | | B x E | ns | ns |
| | | | C x D | ns | ns |
| | | | C x E | ns | 0.36* |
| | | | D x E | ns | ns |
| Faba bean (FB) | Time 2 | Leaf | | | |
| | | | A x C | 0.40* | 0.29* |
| | | | A x E | 0.43* | 0.28* |
| | | | C x E | ns | 0.30* |
| | | Soil | | | |
| | | | A x B | ns | ns |
| | | | A x C | 0.42* | 0.25* |
| | | | A x D | ns | 0.23 |
| | | | A x E | ns | ns |
| | | | B x C | 0.51* | ns |
| | | | B x D | ns | ns |
| | | | B x E | ns | ns |
| | | | C x D | 0.46* | 0.23* |
| | | | C x E | 0.46* | 0.27* |
| | | | D x E | ns | ns |
| Spring barley (SB) | | Leaf | | | |
| | | | A x C | ns | 0.33* |
| | | | A x E | ns | 0.32* |
| | | | C x E | ns | 0.30* |
| | | Soil | | | |
| | | | A x B | ns | 0.36* |
| | | | A x C | ns | 0.26* |
| | | | A x D | ns | ns |
| | | | A x E | ns | ns |

| | | | | | |
|---|---|---|---|---|---|
| | | | B x C | ns | 0.42* |
| | | | B x D | ns | 0.30* |
| | | | B x E | ns | 0.32* |
| | | | C x D | ns | ns |
| | | | C x E | ns | ns |
| | | | D x E | ns | ns |
| Faba bean (FB) | Time 3 | Leaf | | | |
| | | | A x C | 0.56* | ns |
| | | | A x E | ns | ns |
| | | | C x E | ns | ns |
| | | Soil | | | |
| | | | A x B | ns | 0.33* |
| | | | A x C | ns | 0.28* |
| | | | A x D | ns | 0.40* |
| | | | A x E | ns | ns |
| | | | B x C | ns | ns |
| | | | B x D | ns | ns |
| | | | B x E | ns | 0.32* |
| | | | C x D | ns | ns |
| | | | C x E | ns | 0.27* |
| | | | D x E | ns | 0.35* |
| Spring barley (SB) | | Leaf | | | |
| | | | A x C | ns | ns |
| | | | A x E | ns | ns |
| | | | C x E | ns | ns |
| | | Soil | | | |
| | | | A x B | ns | 0.40* |
| | | | A x C | ns | 0.31* |
| | | | A x D | ns | 0.34* |
| | | | A x E | ns | 0.41* |
| | | | B x C | ns | 0.31* |
| | | | B x D | ns | ns |
| | | | B x E | ns | 0.28* |
| | | | C x D | ns | 0.24* |
| | | | C x E | ns | 0.36* |
| | | | D x E | ns | ns |

Significance of test indicated as ***, $p < 0.001$; **, $p < 0.01$; *, $p < 0.05$. The ns denotes not statistically significant and $R^2$ is the proportion of variation explained.
